# Menstrual disorders following COVID-19 vaccination: a review using a systematic search

**DOI:** 10.3389/fdsfr.2024.1338466

**Published:** 2024-01-31

**Authors:** Veerle R. Smaardijk, Rana Jajou, Agnes Kant, Florence P. A. M. van Hunsel

**Affiliations:** ^1^ Netherlands Pharmacovigilance Centre Lareb, Hertogenbosch, Netherlands; ^2^ Department of Clinical Pharmacy and Toxicology, Leiden University Medical Center, Leiden, Netherlands; ^3^ Department of PharmacoTherapy, Epidemiology and Economics, Groningen Research Institute of Pharmacy (GRIP), University of Groningen, Groningen, Netherlands

**Keywords:** COVID-19 vaccines, menstrual disorders, heavy menstrual bleeding, regulatory action, systematic review

## Abstract

**Introduction:**Menstrual disorders are commonly reported after COVID-19 vaccination and heavy menstrual bleeding was added to the product information of the COVID-19 vaccines of Moderna and Pfizer. The aim of this review, using a systematic search, is to provide an overview of available literature on the risk of menstrual disorders after COVID-19 vaccination.

**Methods:** The review was conducted according to the Preferred Reporting Items for Systematic reviews and Meta-Analysis (PRISMA) guidelines. A PubMed search was performed on 15 August 2023, including solely quantitative studies in English and Dutch.

**Results:** A total of 61 studies were included, of which 40 were cross-sectional studies, 18 cohort studies, and three self-controlled case series. Outcomes included a wide variety of menstrual disorders, including changes in cycle length (*n* = 54), changes in the amount of bleeding (*n* = 44), changes in menses length (*n* = 30), changes in the experience of (pre)menstrual pain (*n* = 21), and breakthrough bleeding (*n* = 18). All included studies found a higher percentage of at least one menstrual disorder in the first cycle after different types of COVID-19 vaccination and after different doses.

**Discussion:** In conclusion, evidence suggests that COVID-19 vaccines may cause menstrual changes in women of reproductive age. However, there is a need for more longitudinal studies because of important limitations in the study designs of many of the included studies. Although menstrual changes are short-lived and predominantly mild, it is important for women and healthcare professionals to be informed about these potential adverse reactions and to assess these events in clinical trials on vaccines.

## 1 Introduction

During the COVID-19 pandemic, vaccines development was accelerated (European Medicines Agency, 2020) and in the meantime more than 13 billion doses of COVID-19 vaccines have been administered worldwide (Johns Hopkins Coronavirus Resource Center, 2022). Although vaccines are important to reduce the negative impact of many infectious diseases, adverse reactions after vaccination may occur (Global Advisory Committee on Vaccine Safety GACVSWHO secretariat, 2009; Beatty et al., 2021; Checcucci et al., 2022). Mild adverse reactions such as injection site reactions, fatigue, and headache are expected after vaccination and seen during clinical trials, and were added to the product information of COVID-19 vaccines at the time of (conditional) marketing authorization (Pormohammad et al., 2021; Centers for Disease Control and Prevention, 2022). However, at that time, the safety profile of the COVID-19 vaccines was not completely known and more serious events were also included in the product information of the COVID-19 vaccines after the vaccination campaigns started, such as thrombosis for Vaxzevria (AstraZeneca) and Jcovden (Johnson & Johnson), and peri- and myocarditis for Comirnaty (BioNTech/Pfizer) and Spikevax (Moderna) (European Medicines Agency, 2021b; European Medicines Agency, 2021c; European Medicines Agency, 2021a), hereafter referred to as AstraZeneca, Johnson & Johnson, Pfizer, and Moderna. During the first months after the mass vaccination campaigns in 2021, first reports came in on menstrual changes. These changes were supported by anecdotal reports of menstrual disorders after COVID-19 vaccination on social media (Katz et al., 2022).

Menstrual cycles last on average 26–35 days, with menses lasting about 5 days (Mihm et al., 2011). Menstrual disorders are abnormalities in the menstrual cycle and include changes in the menstrual cycle length, changes in menses length, changes in the amount of bleeding, changes in the experience of (pre)menstrual pain, intermenstrual bleeding, and post-menopausal bleeding (Shapley et al., 2004). Although menstrual disorders are common in women, frequencies of these self-reported outcomes are difficult to estimate (Duijster et al., 2023; Trogstad et al., 2023). Several factors may influence the menstrual cycle, such as lifestyle factors, biological factors, and environmental factors (Campbell et al., 2021). Even small changes in these routine bodily functions related to general health and fertility may have a large adverse impact on multiple aspects of women’s quality of life, including emotional problems and worries, physical complaints, reduced participation in daily activities, and sexual functioning (Matteson and Clark, 2010; Fraser et al., 2015; Nagma et al., 2015; Sveinsdóttir, 2018; Schoep et al., 2019; Campbell et al., 2021; van Galen et al., 2021).

Two reviews and two meta-analyses summarized studies investigating the effect of COVID-19 vaccination on menstrual disorders (Chao et al., 2022; Nazir et al., 2022; Al Kadri et al., 2023). Nazir et al. included 14 studies in their systematic review and found that 52% of the 78,138 included women experienced a menstrual disorder after the COVID-19 vaccine (Nazir et al., 2022). This result was mainly based on cross-sectional studies and they underlined the need for prospective cohort studies. The review by Paik et al. included 11 studies on the incidences and risks of menstruation-related changes after COVID-19 vaccination. They concluded that it remains unclear whether specific groups are more vulnerable to menstrual disturbances after COVID-19 vaccination (Paik and Kim, 2023). The meta-analysis by Chao et al. included four studies comparing menstrual irregularities in vaccinated vs. unvaccinated women (Chao et al., 2022). The pooled OR showed a significant increase of menstrual disorders among vaccinated women (OR = 1.91, 95%CI 1.76–2.07). They recommended that future research should focus on the wide variety of menstrual disorders. A recently conducted meta-analysis by Al Kadri et al. pooled prevalences of sixteen cross-sectional studies on various menstrual disorders after COVID-19 vaccination, of which menorrhagia (24.24%, 95%CI 12.8%–35.6%), oligomenorrhea (22.7%, 95%CI 13.5%–32.0%), and polymenorrhea (16.2%, 95%CI 10.7%–21.6%) were the most common (Al Kadri et al., 2023). Again, a causal relationship could not be established.

The fact that menstrual changes were reported after (newly developed COVID-19) vaccines, is an important issue, since this can contribute to vaccine hesitancy (Muric et al., 2021). Menstrual disorders were prominently reported by women after COVID-19 vaccination. The European Medicines Agency (EMA) estimated that since June 2022 approximately 30% of reports on COVID-19 vaccines received on women could be related to menstrual issues (European Medicines Agency, 2022b), and assessed this potential safety signal (European Medicines Agency, 2022a). Signal detection can be performed in different ways, is multidisciplinary, may use different levels of evidence, and may involve the use of statistical techniques (Harpaz et al., 2022). For example, in the Netherlands, the Pharmacovigilance Centre Lareb monitors spontaneous reports on adverse events after drugs and vaccines, as reported by consumers and health professionals, and discusses potential safety signals with the Dutch Medicines Evaluation Board (CBG-MEB) and, in case of vaccines, also with the National Institute for Public Health and the Environment (RIVM) (Oosterhuis et al., 2023). Regulatory decision making on safety signals for (centrally) registered medicines and vaccines occurs in an European context. For COVID-19 vaccines, after discussion of the signal by the CBG-MEB, signals are forwarded to the Pharmacovigilance Risk Assessment Committee (PRAC). The PRAC is the committee of the EMA and is responsible for assessing and monitoring the safety of human medicines (European Medicines Agency, 2023). Continuous monitoring, early detection, and providing information on possible adverse events is of critical importance to objectively advise the society on the use of drugs and vaccines (Duijster et al., 2023). In other countries, similar systems and organizations for vaccine safety monitoring exist; For instance, the Vaccine Adverse Event Reporting System (VAERS) is the early warning system that monitors the safety of vaccines after they are authorized or licensed for use by the U.S. Food and Drug Administration (FDA). VAERS is part of the larger vaccine safety system in the United States that helps make sure vaccines are safe. The system is co-managed by Centers for Disease Control and Prevention (CDC) and FDA (Harpaz et al., 2022). In October 2022, the PRAC decided that “heavy menstrual bleeding” should be added to the product information of the COVID-19 vaccines of Pfizer and Moderna (European Medicines Agency, 2022a).

The aim of this review using a systematic search strategy is to provide an overview of literature regarding the risk of menstrual disorders after COVID-19 vaccination. Gaps in literature will be discussed, and recommendations for future research will be provided. In addition, we provide a timeline of events and regulatory actions taken regarding safety signals of menstrual disorders following COVID-19 vaccines.

## 2 Materials and methods

This review using a systematic search strategy was developed according to the Preferred Reporting Items for Systematic reviews and Meta-Analysis (PRISMA) guidelines (Page et al., 2021).

### 2.1 Literature search

A literature search protocol was drafted by one reviewer (V.S.) and further refined in cooperation with the project team. We performed the search in PubMed on 15 August 2023 (see Supplementary Figure S1). In addition, a manual search was performed by checking references of published reviews on COVID-19 vaccines and menstrual disorders.

The electronic database search was supplemented by an extensive search on the EMA website including minutes of PRAC meetings, the World Health Organization (WHO) website, the Medicines and Healthcare products Regulatory Agency (MHRA) website, and the FDA website to include all relevant information with regard to the process of this signal assessment. Reports of menstrual disorders after COVID-19-vaccines were extracted from the database of Netherlands Pharmacovigilance Centre Lareb. Supplementary Figure S2 shows a timeline highlighting key events regarding the update of the Summary of Product Characteristics (SmPCs) of Moderna and Pfizer.

### 2.2 Eligibility criteria

Original quantitative studies that examined the association between COVID-19 vaccines and menstrual disorders were included. Articles had to be written in English or Dutch. Studies solely focusing on menstrual disorders after COVID-19 infection were excluded. No restrictions were applied for year of publication, country of publication, study design, type of COVID-19 vaccine, and type of menstrual disorder.

### 2.3 Screening

One reviewer (V.S.) performed the screening on titles and abstract in EndNote, based on the established criteria. A random selection of twenty included and excluded citations was screened by a second reviewer (R.J.). Any discrepancies between the two reviewers were discussed. Next, the full-text screening was performed by one reviewer (V.S.).

### 2.4 Data-extraction

Relevant data from included studies were extracted by one reviewer (V.S.) using a customized data-extraction form. The form was pilot-tested using five included papers and adapted in collaboration with the project team. In the final data-extraction form, the following variables were extracted: first author, year of publication, country, type of study design, type(s) of vaccine, number of analyzed people, age (mean, median, and/or range), data source/measurement methods, outcome(s), and main findings. We also listed additional notes about the study, such as important recommendations or limitations of the study. Findings from the included studies were presented using a narrative synthesis.

## 3 Results

### 3.1 Literature search

**FIGURE 1:**
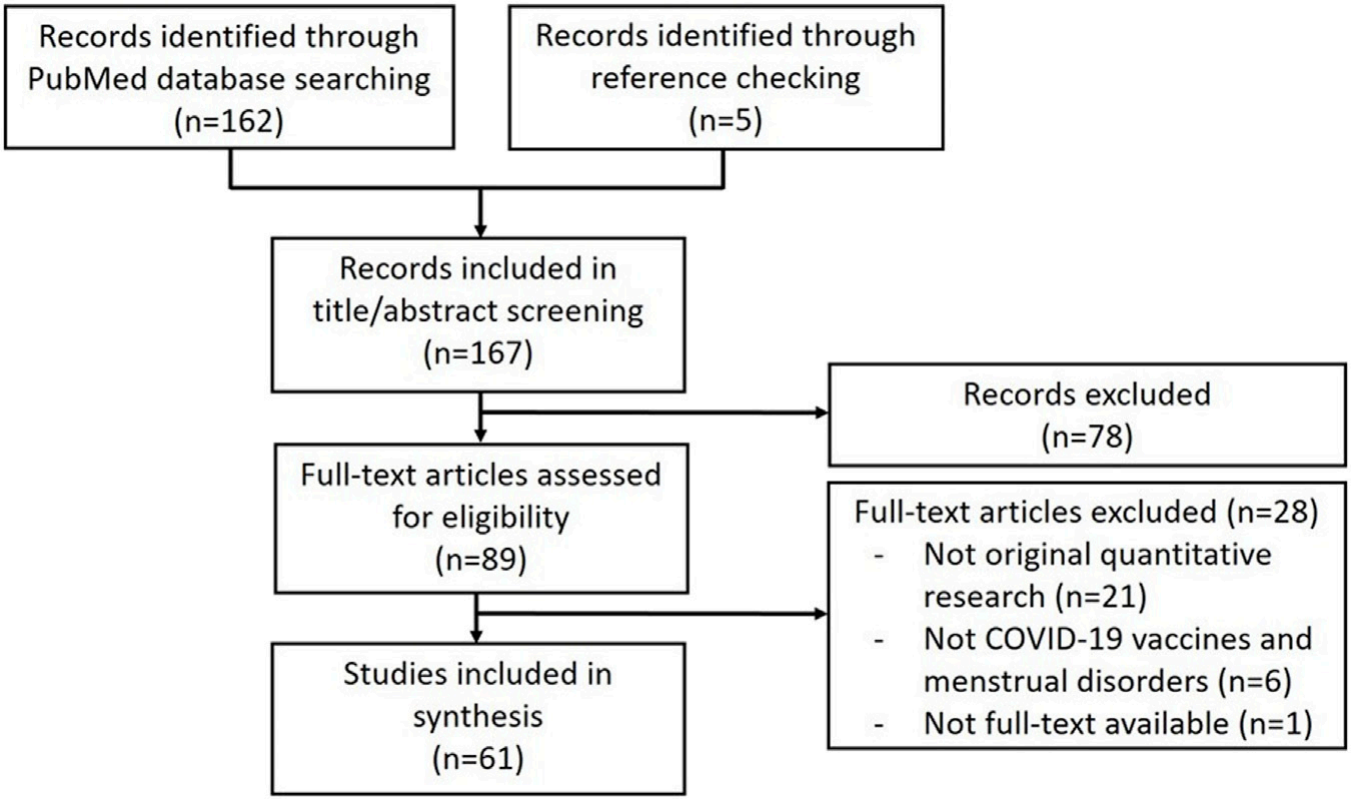
Flow chart of the screening process.

The PubMed search resulted in 162 records. We found five additional eligible records through our manual search. Of the 167 records screened on title/abstract, 89 were included in the full-text screening. After full-text screening, 28 studies were excluded because of a non-quantitative study design (n = 21) or an irrelevant topic (n = 6). We were unable to retrieve the full-text version of one paper. In total, 61 studies were included in our review (Alghamdi et al., 2021; Abdollahi et al., 2022; Edelman et al., 2022a; Akarsu, 2022; Al-Mehaisen et al., 2022; Alahmadi et al., 2022; Aldali et al., 2022; Alvergne et al., 2022; Amer et al., 2022; Anjorin et al., 2022; Baena-García et al., 2022; Barabás et al., 2022; Edelman et al., 2022b; Dabbousi et al., 2022; Dar-Odeh et al., 2022; Dellino et al., 2022; El-Shitany et al., 2022; Farhat et al., 2022; Farland et al., 2022; Gibson et al., 2022; Issakov et al., 2022; Laganà et al., 2022; Lee et al., 2022; Mínguez-Esteban et al., 2022; Morsi et al., 2022; Muhaidat et al., 2022; Namiki et al., 2022; Qashqari et al., 2022; Rodríguez Quejada et al., 2022; Rogers et al., 2022; Sarfraz et al., 2022; Sualeh et al., 2022; Taşkaldıran et al., 2022; Velasco-Regulez et al., 2022; Wang et al., 2022; Wong et al., 2022; Al-Furaydi et al., 2023; Caspersen et al., 2023; Chiang et al., 2023; Kajiwara et al., 2023; Lessans et al., 2023; Matar et al., 2023; Trogstad et al., 2023; Cheng et al., 2022; Velasco-Regulez et al., 2022; Wang et al., 2022; Wong et al., 2022; Woon and Male, 2022; Zhang et al., 2022; Alvergne et al., 2023a; Alvergne et al., 2023b; Bisgaard Jensen et al., 2023; Darney et al., 2023; Trogstad et al., 2023; Duijster et al., 2023; Farah et al., 2023; Filfilan et al., 2023; Hariton et al., 2023; Ljung et al., 2023; Mohr-Sasson et al., 2023; Qazi et al., 2023; Rastegar et al., 2023; Saleh Alzahrani et al., 2023; Wali et al., 2023; Wesselink et al., 2023). Figure 1 shows a flow chart of the selection process.

Of the 61 included studies, 40 were cross-sectional studies, 18 cohort studies, and three self-controlled case series (two as part of a larger cohort study). One study was published in 2021, the remaining in 2022 (n = 42, 69%) and 2023 (n = 18, 30%). A substantial number of studies was performed in Saudi Arabia (n = 13, 21%). The majority of studies used self-reported (online) questionnaires to assess the presence of menstrual disorders (n = 52, 85%), often spread through social media, whereas some used a menstrual cycle tracking app (n = 7, 11%). Most studies excluded participants younger than 18 years. Three studies only included female adolescents aged 12–15, 12–16 or 12–17 years (Aldali et al., 2022; Caspersen et al., 2023; Mohr-Sasson et al., 2023). The study characteristics of the 61 included studies are shown in Supplemental File S3.

In most studies, the Pfizer vaccine was the most administered vaccine (n = 48, 79%). Other administered vaccines were Moderna, Johnson & Johnson, AstraZeneca, Sinopharm, Sputnik V, Covaxin, Sinovac/Coronavac, Turkovac, and Coviran Barkat. Different results were found regarding menstrual disorders after specific COVID-19 vaccine brands. Some studies did not find differences between the vaccine brands AstraZeneca, Johnson & Johnson, Moderna, and Pfizer (Alvergne et al., 2022; Muhaidat et al., 2022; Qashqari et al., 2022; Rogers et al., 2022; Alvergne et al., 2023b; Darney et al., 2023; Saleh Alzahrani et al., 2023; Wali et al., 2023), whereas others found that Pfizer (Wong et al., 2022; Al-Furaydi et al., 2023; Duijster et al., 2023; Filfilan et al., 2023), Moderna (Alahmadi et al., 2022; Trogstad et al., 2023), or AstraZeneca (Filfilan et al., 2023) resulted in a higher rate of menstrual changes compared to other COVID-19 vaccines.

### 3.2 COVID-19 vaccines and menstrual disorders

Outcomes included a wide variety of menstrual disorders, including changes in cycle length (n = 54), changes in the amount of bleeding (n = 44), changes in menses length (n = 30), changes in the experience of (pre)menstrual pain (n = 21), and breakthrough bleeding (n = 18). Two studies did not specify the type of menstrual disorders (Anjorin et al., 2022; Dar-Odeh et al., 2022). However, the outcomes are ambiguous. For example, “menstrual irregularities” might comprise more than only an increase or decrease in cycle length, and the terms “heavy menstrual bleeding” and “prolonged menses length” might be interpreted interchangeably or combined (Farland et al., 2022). Changes in menses length can also be described as changes in inter-menstrual duration.

All included studies found a higher percentage of at least one menstrual disorder in the first cycle after different types of COVID-19 vaccination and after different doses. All included cohort studies found a positive association between COVID-19 vaccines and one or more menstrual disorders. These studies also mentioned that these disturbances were transient. Overall, menstrual disorders were predominantly mild and in most cases the menstrual cycle returned to normal within several months or by the time the second dose was given (Alahmadi et al., 2022; Edelman et al., 2022b; Farland et al., 2022; Gibson et al., 2022; Laganà et al., 2022; Rogers et al., 2022; Taşkaldıran et al., 2022; Wang et al., 2022; Woon and Male, 2022; Alvergne et al., 2023a; Al-Furaydi et al., 2023; Darney et al., 2023; Trogstad et al., 2023; Wesselink et al., 2023).

Several studies investigated factors associated with an increased risk of menstrual changes after COVID-19 vaccination. One study found that heavy menstrual bleeding after vaccination was associated with a history of prolonged and heavy menses (Issakov et al., 2022). Other comorbidities including endometriosis, hypertension, menorrhagia, fibroids, adenomyosis, thyroid disorders, and Polycystic Ovary Syndrome (PCOS) were also significantly related factors (Lee et al., 2022; Muhaidat et al., 2022; Farah et al., 2023; Saleh Alzahrani et al., 2023). Although the use of non-hormonal intrauterine devices was positively correlated with excessive bleeding in one study (Issakov et al., 2022; Alvergne et al., 2023b) found that hormonal contraception decreased the risk of reporting menstrual changes by 50%, Farland et al. (Farland et al., 2022) found that using any hormonal medication decreased the risk of menstrual cycle changes by 27% (OR = 0.73; 95%CI 0.49–1.08), and (Bisgaard Jensen et al., 2023) found lower odds of reporting any menstrual change (OR = 1.71; 95%CI 0.65–0.78). Two studies did not find an association between hormonal contraceptives and menstrual abnormalities (Muhaidat et al., 2022; Duijster et al., 2023). Other significant associated factors that increased the risk were age (Lee et al., 2022; Morsi et al., 2022; Farah et al., 2023), greater body mass index (Farland et al., 2022), number of children (Muhaidat et al., 2022), marital status (Morsi et al., 2022; Muhaidat et al., 2022), smoking (Alvergne et al., 2023b), education level (Farah et al., 2023), being Hispanic of Latinx (Lee et al., 2022), and high self-reported perceived stress levels (Farland et al., 2022; Bisgaard Jensen et al., 2023). A history of COVID-19 infection decreased the risk in the study by Farland et al. (OR = 0.58; 95%CI 0.32–1.04) (Farland et al., 2022), while Alvergne et al. and Bisgaard Jensen et al. found higher risks for this subgroup (PR = 1.46, 95%CI 1.22–1.75, OR = 2.17, 95%CI 1.40–3.35, respectively) (Alvergne et al., 2023b; Bisgaard Jensen et al., 2023). Trogstad et al. did not find significant results in their additional analyses on concurrent medical conditions and concomitant medication (Trogstad et al., 2023).

### 3.3 COVID-19 vaccination and menstrual cycle length

Changes in menstrual cycle length after COVID-19 vaccination was the outcome most assessed (n = 54, 89%). The majority of studies found a statistically significant difference on this outcome between vaccinated and unvaccinated individuals. A globally conducted cohort study by Edelman et al. including 19,622 individuals (of whom 14,936 vaccinated) found an increase in cycle length of less than 1 day for both doses, compared to unvaccinated individuals (0.71 days increase (99.3%CI 0.47–0.96) for first dose; 0.56 days increase (99.3%CI 0.28–0.84) for second dose) (Edelman et al., 2022b). Another large cohort study by Gibson et al. with a sample size of 9,652 participants (of whom 8,485 vaccinated) found similar results (first dose 0.50 days increase, 95%CI 0.22–0.78 and second dose 0.39 days increase, 95%CI 0.11–0.67) (Gibson et al., 2022). Trogstad et al. found significant differences on both shorter and longer intervals after first and second doses (RRs ranging from 1.24 to 1.57) (Trogstad et al., 2023). Bisgaard Jensen et al. mentioned that change in menstrual cycle length was the most frequently reported change (9% longer menstrual cycles and 7% shorter menstrual cycles (Bisgaard Jensen et al., 2023). In the study by Duijster et al., amenorrhoea/oligomenorrhoea was also the most reported menstrual outcome (33% of 24,090 spontaneous reports) (Duijster et al., 2023). Caspersen et al. found a longer interval in girls aged 12–15 years (RR = 1.15, 95%CI 1.05–1.27), and in girls aged 14–15 years (RR = 1.17, 95%CI 1.05–1.31). This study also found an increased risk on shorter intervals in girls aged 12–15 years (RR = 1.19, 95%CI 1.07–1.32), and girls aged 14–15 years (RR = 1.18, 95%CI 1.05–1.33) (Caspersen et al., 2023). Muhaidat et al. found an increase in cycle length from 27 ± 6 days prior to vaccination to 28.1 ± 10 days after being vaccinated (*p* < 0.001) (Muhaidat et al., 2022). Significant changes were also reported by Velasco-Regulez et al. (increase in median cycle length of 0.5 (0.0–1.0) days (*p* < 0.005)) (Velasco-Regulez et al., 2022), and Wang et al., who found that vaccinated women had a higher risk of an increased cycle length compared to unvaccinated women (OR = 1.48; 95%CI 1.00–2.19) (Wang et al., 2022). Woon et al. showed in their prospective cohort study that either dose of the COVID-19 vaccine was associated with a delay to the following period (2.3 days after dose 1 (*p* = 0.0045); 1.3 days after dose 2, *p* = 0.041) (Woon and Male, 2022). Alvergne et al. found a significant increase in the menstrual cycle length of 2.3 days after the first dose, and 1.3 days after the second dose (Alvergne et al., 2022).

### 3.4 COVID-19 vaccination and the amount of menstrual bleeding

The second most investigated outcome was the association between COVID-19 vaccines and alterations in the amount of bleeding (n = 44, 72%). Most studies found a higher percentage of people reporting that their next period was heavier than normal after receiving the first and/or second dose of the COVID-19 vaccine. Most of these studies found that the menstrual flow returned to normal within several months (Laganà et al., 2022; Muhaidat et al., 2022; Darney et al., 2023; Trogstad et al., 2023), whereas another study reported that this alteration could last more than 5 months (Mínguez-Esteban et al., 2022). Caspersen et al. found in their cohort study that the risk of heavier menstrual bleeding was higher in the menstrual cycle after vaccination compared to the cycle before vaccination (RR = 1.61, 95% CI 1.43–1.81) (Caspersen et al., 2023). In this cohort, 99.9% received the Pfizer-vaccine. The cohort study by Trogstad et al. found a relative risk of more heavy bleeding of 1.90 (95% CI 1.69–2.13) for the first dose with Pfizer or Moderna, while the RR was 1.84 (95% CI 1.66–2.03) for the second dose (Trogstad et al., 2023). Zhang et al. found a relatively low percentage of reports of menorrhagia (0.2%), compared to other menstrual outcomes after COVID-19 vaccination in their study (Zhang et al., 2022). Alvergne et al. and Wesselink et al. did not find an association between COVID-19 vaccination and menstrual flow (Alvergne et al., 2022; Wesselink et al., 2023).

### 3.5 COVID-19 vaccination and menses length

Thirty studies (49%) investigated changes in menses length after COVID-19 vaccination. Muhaidat et al. found an increase from 6 ± 0.03 days pre-vaccine to 6.5 ± 0.1 post-vaccine (*p* < 0.001) (Muhaidat et al., 2022). Caspersen et al. compared the menstrual cycle after vaccination and the cycle before vaccination in girls aged 12–15 years and found a RR for increased menses length of 1.40 (95% CI 1.23–1.60) (Caspersen et al., 2023). Eight percent of the vaccinated individuals in the study of Barabas et al. suffered from prolonged bleeding lasting for more than 2 weeks (Barabás et al., 2022). Lastly, Trogstad et al. found a RR of 1.46 (95% CI 1.31–1.61) after the first dose, and a RR of 1.71 (95% CI 1.55–1.89) after the second dose on prolonged bleeding (Trogstad et al., 2023). Several other studies mentioned that they did not find significant results on COVID-19 vaccination and menses length (Edelman et al., 2022a; Edelman et al., 2022b; Velasco-Regulez et al., 2022; Darney et al., 2023; Wesselink et al., 2023).

### 3.6 COVID-19 vaccination and the experience of (pre)menstrual pain

Heavier or less menstrual pain or cramps during the next period after COVID-19 vaccination was assessed by 21 (34%) studies. Trogstad et al. found a significant increase in the percentage of people experiencing more pain between the last cycle before vaccination and the first cycle after vaccination (first dose: 11.4% vs. 14.6%, RR = 1.24 (95% CI 1.24–1.47), second dose: 9.8% vs. 16%, RR = 1.62 (95% CI 1.49–1.77)) (Trogstad et al., 2023). Caspersen et al. also found a significant increase on stronger period pains (RR = 1.14, 95% CI 1.04–1.26 for girls aged 12–15 years), and RR = 1.14 (95% CI 1.02–1.27) for girls aged 14–15 years (Caspersen et al., 2023; Caspersen et al., 2023). Several other studies also found increased percentages on menstrual cramps after vaccination (Alahmadi et al., 2022; Farland et al., 2022; Morsi et al., 2022; Qashqari et al., 2022; Duijster et al., 2023; Filfilan et al., 2023; Qazi et al., 2023). On the other hand, Morsi et al. also found that 11% reported a decrease in the severity of pain (Morsi et al., 2022). Two studies observed no significant changes in the percentages of menstrual pain intensity after vaccination (Velasco-Regulez et al., 2022; Wesselink et al., 2023).

### 3.7 COVID-19 vaccination and breakthrough bleeding

Eighteen studies (30%) investigated the association between COVID-19 vaccination and breakthrough bleeding, spot bleeding, postmenopausal bleeding and/or premenopausal bleeding. A large population-based cohort study including 2,580,007 vaccinated Swedish women found the highest risk for postmenopausal bleeding after the third dose between 1 and 7 days (HR 1.28, 95%CI 1.01–1.62) and between 8 and 90 days (HR = 1.25, 95%CI 1.04–1.50) (Ljung et al., 2023). Trogstad et al. found a RR of 1.09 (95%CI 1.01–1.17) after the first dose, and a RR of 1.49 (95%CI 1.37–1.62) after the second dose (Trogstad et al., 2023). Other studies reported 1% (Farah et al., 2023), 2.4% (Taşkaldıran et al., 2022), 16% (Zhang et al., 2022), and 19% (Duijster et al., 2023) of women experiencing intermenstrual bleeding after vaccination. (Caspersen et al., 2023). did not find an association between COVID-19 vaccination and spot bleeding (Caspersen et al., 2023; Lee et al., 2022) reported that 66% of postmenopausal women experienced a breakthrough bleeding. This outcome was significantly associated with age, systemic vaccine side effects (fever and/or fatigue), history of pregnancy or birth, and ethnicity (Lee et al., 2022).

### 3.8 Timing of vaccination

Discrepancies exist between studies with regard to the influence of timing of vaccination on menstrual disorders. Some studies did not find an effect of timing on the flow or the next period (Alvergne et al., 2022; Woon and Male, 2022; Darney et al., 2023), whereas others stated that the risk of menstrual disturbances was higher in specific phases of the menstrual cycle (Gibson et al., 2022; Velasco-Regulez et al., 2022; Caspersen et al., 2023; Kajiwara et al., 2023). One study stated that COVID-19 vaccination has a larger impact on menstrual regularity when both doses are given within the same menstrual cycle (Edelman et al., 2022a; Gibson et al., 2022; Velasco-Regulez et al., 2022; Kajiwara et al., 2023; Gibson et al., 2022) found that vaccination during the estimated follicular phase was associated with an increased menstrual cycle length in first-dose cycles (0.97 days, 95% CI 0.53–1.42) or second-dose cycles (1.43 days, 95% CI 1.06–1.80) of mRNA vaccines or the Johnson & Johnson vaccine (2.27 days, 95% CI 1.04–3.50), compared with pre-vaccination cycles). In their study, mRNA vaccination during the luteal phase was associated with a decreased menstrual cycle length (−0.97 days, 95% CI –1.39 to −0.55). (Velasco-Regulez et al., 2022). also found a significant increase in median cycle length of 1 (0.0–1.0) days in people vaccinated during the follicular phase. However, they did not find changes in people vaccinated during the luteal phase. Edelman et al. stated that COVID-19 vaccination has a larger impact on menstrual regularity when both doses are given within the same menstrual cycle, with a mean delay of 2.32 days (98.75% CI 1.59–3.04) to their next period (Edelman et al., 2022a; Kajiwara et al., 2023) also found significantly increased menstrual cycle lengths in participants who received vaccinations twice within a single menstrual cycle.

## 4 Discussion

### 4.1 Summary of evidence

This review synthesized the body of evidence on menstrual disorders following COVID-19 vaccination. In addition, we summarized data on key events regarding the signal detection process. Evidence of 61 primary studies, including 18 cohort studies, shows that COVID-19 vaccines may cause menstrual changes in women of reproductive age. Although the menstrual changes are short-lived and predominantly mild, it is important for women and healthcare professionals to be informed about these potential adverse reactions.

For the association between menstrual disorders and COVID-19 vaccines it should be considered that the menstrual cycle itself is a complex, coordinated sequence of events involving the hypothalamus, anterior pituitary, ovary, and endometrium. Menstrual disorders such as heavy menstrual bleeding in a female of reproductive age can be related to the disturbance of normal hormonal, physiological mechanism, or female anatomic abnormalities (Oertelt-Prigione, 2012). As a mechanism for the biological link between COVID-19 vaccination and menstrual abnormalities, the systemic immune response after vaccination might interfere in many pathways that are involved in the menstrual cycle (Wong et al., 2022). These include, for example, hormonal and inflammatory pathways. Also, it has been suggested that the number of certain immune cells as well as their activity differs between the first and second part of the menstrual cycle (Oertelt-Prigione, 2012).

### 4.2 Research gaps

The most important limitation of many included studies is the cross-sectional study design, which limits the possibility to investigate a causal relationship between the COVID-19-vaccines and menstrual disorders (Sharp et al., 2022). Second, the majority of the included studies reported only descriptive statistics, and did not use a control group. Since menstrual disorders are common in the general population, this is an important gap in previously conducted research in this area (European Medicines Agency, 2022b). Third, most studies excluded women younger than 18 years. Since the median age of menarche is 11.9 years (Martinez, 2020), it would be interesting to examine menstrual outcomes in vaccinated female adolescents as well. Fourth, most studies were performed in high-income countries with high access to healthcare access and resources to track and report menstrual disorders, which limits generalizability of the results to other countries (Nazir et al., 2022). Due to the fact that talking openly about menstruation is a taboo in some countries as well as less reporting to spontaneous reporting systems, the number of menstrual disorders might be underestimated in several studies (Sarfraz et al., 2022). The fifth limitation of the included studies is that many studies distributed the questionnaire via different social media platforms (e.g., Facebook, WhatsApp, LinkedIn, and Twitter). This may have led to selection bias with a higher inclusion of younger women and women who feel that the vaccine affected their menstruation (Issakov et al., 2022). However, social media platforms are modern instruments to quickly reach the whole country and appropriate age groups (European Medicines Agency, 2022b). Moreover, several studies were performed after vaccination took place and after an increase of menstrual irregularities reported in the media, which may have led to an overestimation of actual incidence numbers (Oertelt-Prigione, 2012). Lareb found clear peaks of received reports when menstrual disorders were discussed in the media (Netherlands Pharmacovigilance Centre Lareb, 2022), although this seems to correlate with the start date of age groups that were eligible to be vaccinated in the Dutch vaccination program. Sixth, because of the retrospective design of many includes studies, recall bias might have occurred. At last, the menstrual disorders are mainly non-serious, with short-term duration, and self-reported without confirmation of a healthcare professional (Sharp et al., 2022). However, many women do not visit their gynecologist for minor menstrual disturbances and physicians can only confirm what the woman told him. Possibilities to medically confirm transient menstrual disturbances are limited, which means that medical confirmation does not increase the credibility of the self-reported cases (European Medicines Agency, 2022b; European Medicines Agency, 2022c).

### 4.3 Implications for further research

In addition to the aforementioned research gaps and implications for further research to cover these limitations, we recommend to further investigate differences between vaccine types and number of doses. We also recommend to examine differences between groups who might be more vulnerable to develop menstrual disorders, including older women, women with a higher body mass index (BMI), women with gynecological disorders, and women using hormonal contraceptives. In addition, it would be interesting to investigate the long-term effects of COVID-19 vaccines on the menstrual cycle, and to examine the biological mechanism of COVID-19 vaccines and menstrual disorders, since the pathological pathway is yet not completely understood. Menstrual changes, even small or temporary, may have a high impact on women’s daily life and risks need to be addressed by healthcare professionals during vaccination counseling and investigated in clinical trials on vaccination (Al Kadri et al., 2023; Alvergne, 2023). To gain more knowledge on menstrual disorders after COVID-19 vaccination and to investigate optimal timing of the menstrual cycle with regard to vaccine efficacy and reduced adverse effects, women should be screened on disturbances before vaccination and menstrual changes should be monitored over time (Al Kadri et al., 2023; Alvergne, 2023; Trogstad et al., 2023).

### 4.4 Study limitations

This review using a systematic search has some limitations. First, we did not perform a risk of bias assessment of the included studies. Due to this, studies of relatively poor study designs were accepted. However, we extracted relevant data including additional notes such as flaws in the study design or other limitations of the study. Second, literature screening and data-extraction was performed by one person, which limits the reliability of the results. Third, we only searched in one database (Pubmed). However, we reference-checked published studies and all relevant information from regulator websites and published reports with regard to the process of signal assessment, which resulted in five more studies.

## 5 Conclusion

This review, using a systematic search strategy, identified a wide range of studies investigating a variety of menstrual disorders after COVID-19 vaccination. Although there were important limitations in the study designs of many of the included studies and there is a need for more longitudinal studies, evidence suggests that COVID-19 vaccines may cause menstrual changes in women of reproductive age. Because of the high impact of menstrual disorders on women’s quality of life, menstruation-related adverse reactions should be investigated when developing vaccines. In this way, women can make well-informed decisions about taking a vaccine.

## Data Availability

The original contributions presented in the study are included in the article/Supplementary Material, further inquiries can be directed to the corresponding author.
