# Supplementary material for: Menstrual disorders following COVID-19 vaccination: a review using a systematic search
Source: Front Drug Saf Regul. 2024 Jan 31;4:1338466. doi: 10.3389/fdsfr.2024.1338466 (PMC12443100; doi:10.3389/fdsfr.2024.1338466)
Supplement: Supplementary file 1 [file Table1.DOCX]

Supplementary Material

# SUPPLEMENTAL FILES

## Supplemental File S1. Literature search in PubMed

| **COVID-19** | "COVID-19” [MeSH] OR “COVID-19” OR “COVID19” OR “COVID2019” OR “COVID 2019” OR “coronavirus” OR “SARS-CoV-2” [MeSH] OR SARS-CoV-2 OR "SARSCoV2" OR "SARS-CoV2" OR "2019nCoV" OR "2019-nCoV" OR “nCoV-2019” OR "2019 coronavirus" OR "2019 corona virus" OR "coronavirus disease 2019" |
| --- | --- |
|  | *AND* |
| **Vaccines** | “COVID-19 vaccines” [MeSH] OR “COVID-19 vaccine” OR “COVID-19 vaccines” OR “mRNA vaccines” [MeSH] OR mRNA vaccin OR “Vaccination” [MeSH] OR vaccination OR "mRNA-1273 vaccine" OR "mRNA vaccine" OR "mRNA COVID-19 vaccines" OR "BNT162 vaccine" OR "BNT162b2" OR "BNT162" OR "Spikevax" OR "Comirnaty" OR “ChAdOx1 nCoV-19” [MesH] OR “ChAdOx1 nCoV-19” OR “Immunization” [MeSH] OR immun* OR "Immunization Programs"[Mesh] OR “immunization program” OR “immunization programs” OR "Injections"[Mesh] OR inject* OR vaccin* |
|  | *AND* |
| **Menstruation disorders** | “Menstruation Disturbances” [MeSH] OR “menstruation disturbance” OR “Menstrual Cycle” [MeSH] OR “menstrual cycle” OR “irregular menstrual cycle” OR “menstrual” OR “Menorrhagia” [MeSH] OR “menorrhagia” OR “Metrorrhagia” [MeSH] OR “metrorrhagia” OR “Menarche” [MeSH] OR “menarche” OR “Menstruation” [MeSH] OR “menstruation” |

## Supplemental File S2. Signal assessment procedure

Supplemental Figure 1 shows a timeline highlighting key events regarding the update of the Summary of Product Characteristics (SmPCs) of Moderna and Pfizer.

*August 2021*

In August 2021, EMA’s PRAC assessed scientific literature on reported cases of menstrual disorders occurring after COVID-19 vaccination. Based on the assessment of all data by the Danish Medicines Agency (DMA), i.e. literature, approximately 2,800 reported cases in Denmark, and detailed analyses on characteristics of these cases, the PRAC concluded that there was no evidence for a causal relationship between the vaccines and menstrual disorders [1]. Menstrual disorders were considered common in the general population and were in the reported cases most likely caused by other events, such as stress or underlying medical conditions. Moreover, no potential mechanism could be identified. The Norwegian Medicines Agency (NoMA) also raised awareness for prioritization of this signal, based on the reports they received (over 50 reports of various types of menstrual disorders by June 2021) [2-5]. The Medicines Agencies continued to monitor the potential safety issue.

*September 2021*

So far, the MHRA received over 30,000 reports of menstrual changes following COVID-19 vaccination in the British Yellow Card surveillance system and they also evaluated these reports [6, 7]. Similar to the PRAC, they concluded that there is no link since the number of reports is low relative to the general prevalence of menstrual disorders and the number of people who received COVID-19 vaccines. They also noticed that menstrual changes were mostly transient. They continued to review the reports of the Yellow Card scheme [6]. However, the need to perform research to explore the link between COVID-19 vaccines and menstrual changes increased. Researchers stated that comparisons between vaccinated and unvaccinated people should be investigated, and cases of menstrual problems were probably underreported in the pharmacovigilance systems since women might be ashamed of their events, might not have thought the events were related to the COVID-19 vaccine, or might not have been encouraged by their healthcare professional to report their adverse event in the reporting system [7, 8]. The National Institutes of Health (NIH) has awarded supplemental grants to five American institutions to explore potential links and to investigate potential mechanisms [9].

*October 2021*

The first study on menstrual disorders after COVID-19 vaccination was published in October 2021 [10]. This cross-sectional study, conducted in Saudi Arabia, analyzed more than 4,000 people vaccinated with Pfizer or AstraZeneca. In total, 0.98% of the female participants reported a delay in their menstrual cycle or an increase in bleeding or pain after the first and second dose of the Pfizer COVID-19-vaccine, compared to 0.68% after the first dose of the AstraZeneca-vaccine.

*December 2021*

In December 2021, The Netherlands Pharmacovigilance Centre Lareb published a signal covering menstrual disorder reports [11, 12]. From January 6, 2021 (start of the National COVID-19 immunization campaign) until December 1, 2021, Lareb received a total of 17,735 individual case reports of menstrual disorders and postmenopausal blood loss after AstraZeneca, Johnson&Johnson, Moderna and Pfizer (see Supplemental Figure 2). With regard to heavy menstrual blood loss, this number was 4,537 (2,481 after the first dose, 2,056 after the second dose). Of these, 26 (0.6%) were serious reports. At the time of reporting, 26% were recovered/resolved with a mean duration of 13 days. The highest reporting rates were after Johnson&Johnson in the age category 20-45 years (134.5 per 100,000 vaccinations), followed by 90.1 reports per 100,000 vaccinations after the first dose of Pfizer in the same age category. Overall, the highest reporting rates were after Johnson&Johnson (98.3 reports per 100,000 vaccinations), followed by Moderna (51.4 reports per 100,000 first dose vaccinations, 62.5 reports after second dose vaccinations), Pfizer (36.8 reports per 100,000 first dose vaccinations, 37.0 reports after second dose vaccinations), and AstraZeneca (9.1 reports per 100,000 first dose vaccinations, 9.5 reports after second dose vaccinations). Lareb stated that it is plausible that the COVID-19 vaccines caused the menstrual disorders.

*January 2022*

In January 2022, two large studies on menstrual disorders after COVID-19 vaccination were published [13, 14]. The cohort study by Edelman et al. did not include heavy menstrual bleeding as an outcome, but solely focused on menstrual cycle length and menses length. In 3,959 Americans between 18 and 45 years, of whom 2,403 vaccinated, a less than 1-day change difference in cycle length between vaccinated and unvaccinated people was found (first dose 0.64 days, 98.75%CI 0.27-1.01; second dose 0.79 days, 98.75%CI 0.40-1.18) [13]. The self-controlled case series study by Trogstad et al. included a broad spectrum of menstrual disorders, including more heavy bleeding than usual. They analyzed 5,688 Norwegian women between 18 and 30 years and found a relative risk of 1.90 (95%CI 1.69-2.13) of more heavy bleeding than usual for vaccinated women (first dose Moderna of Pfizer) compared to unvaccinated women. For the second dose, the RR was 1.84 (95%CI 1.66-2.03). Menstrual disturbances returned to normal within approximately two months [14].

*February 2022*

The findings from the literature and the increased number of spontaneous reports led to further assessment of heavy periods or amenorrhea following COVID-19 vaccination by the PRAC in February 2022. At this point, it was still not clear whether there is a causal association between the COVID-19 vaccines (Moderna and Pfizer) and the reports of heavy menstrual bleeding or amenorrhea. The PRAC decided to request an in-depth evaluation of available data from spontaneous reporting systems (EudraVigilance), cases reported during clinical trials, and data from the literature [15]. Four months later, the committee continued the assessment of the safety signal and requested from the marketing authorization holders an updated cumulative review of the cases of heavy periods. With regard to amenorrhea, the PRAC concluded that there was insufficient data to establish a causal link between Moderna or Pfizer and the absence of menstruation [16].

*March 2022*

Until March 2022, The WHO global database VigiBase received 186,962 reports of menstrual disorders, of which 47,486 of heavy menstrual bleeding. Of these, 4,508 were serious reports. Most of the menstrual reports were associated with the Pfizer vaccine [12]. In the meantime, Lareb received over 27,000 cases of various menstrual disorders. The Johnson&Johnson vaccine had the highest reporting rates compared to the other vaccines (523 reports per 100,000 vaccinations) [17]. Amenorrhea/oligomenorrhea was the most reported menstrual disorder (33%), followed by heavy menstrual bleeding (29%) and irregular bleeding (23%). No large differences between vaccine brands were found. An overview of menstrual disorders after COVID-19 vaccinations reported to Lareb and possibly related factors are described in the study of Duijster et al. [17].

*October-November 2022*

In October 2022, the PRAC has finalized the assessment of heavy menstrual bleeding and recommended that this side effect should be added to the product information of the COVID-19 vaccines Pfizer and Moderna. Based on worldwide reports of heavy menstrual bleeding after first, second and booster doses, the PRAC concluded that there is at least a reasonable possibility that the occurrence of heavy menstrual bleeding is casually associated with these vaccines [18]. ‘A reasonable possibility of a causal association’ is a criterion for SmPC update. Most of the reviewed cases had transient and non-serious complaints. The frequency category assigned to the adverse event is ‘not known’, since frequencies from spontaneous reports of suspected side effects are difficult to estimate. At this point, cases of heavy menstrual bleeding were still monitored and healthcare professionals and patients were encouraged to continue to report these side effects to the national authorities. A pathophysiological mechanism was not yet understood [18, 19]. The MHRA has also continued to review reports of menstrual disorders following COVID-19 vaccination in the UK. At the end of November 2022, a total of 51,695 suspected reactions relating to a variety of menstrual disorders has been reported in 40,327 individual Yellow Card reports. Their review did not support a link between COVID-19 vaccines and other menstrual disorders [20].

*November-December 2022*

On November 25, 2022, the product information of Moderna and Pfizer was amended by adding the following text: ‘Reproductive system and breast disorders: Heavy menstrual bleeding. Frequency: not known (cannot be estimated from the available data)’ [2, 3, 21, 22]. A footnote stating that ‘most cases appeared to be non-serious and temporary in nature’ was added.

**
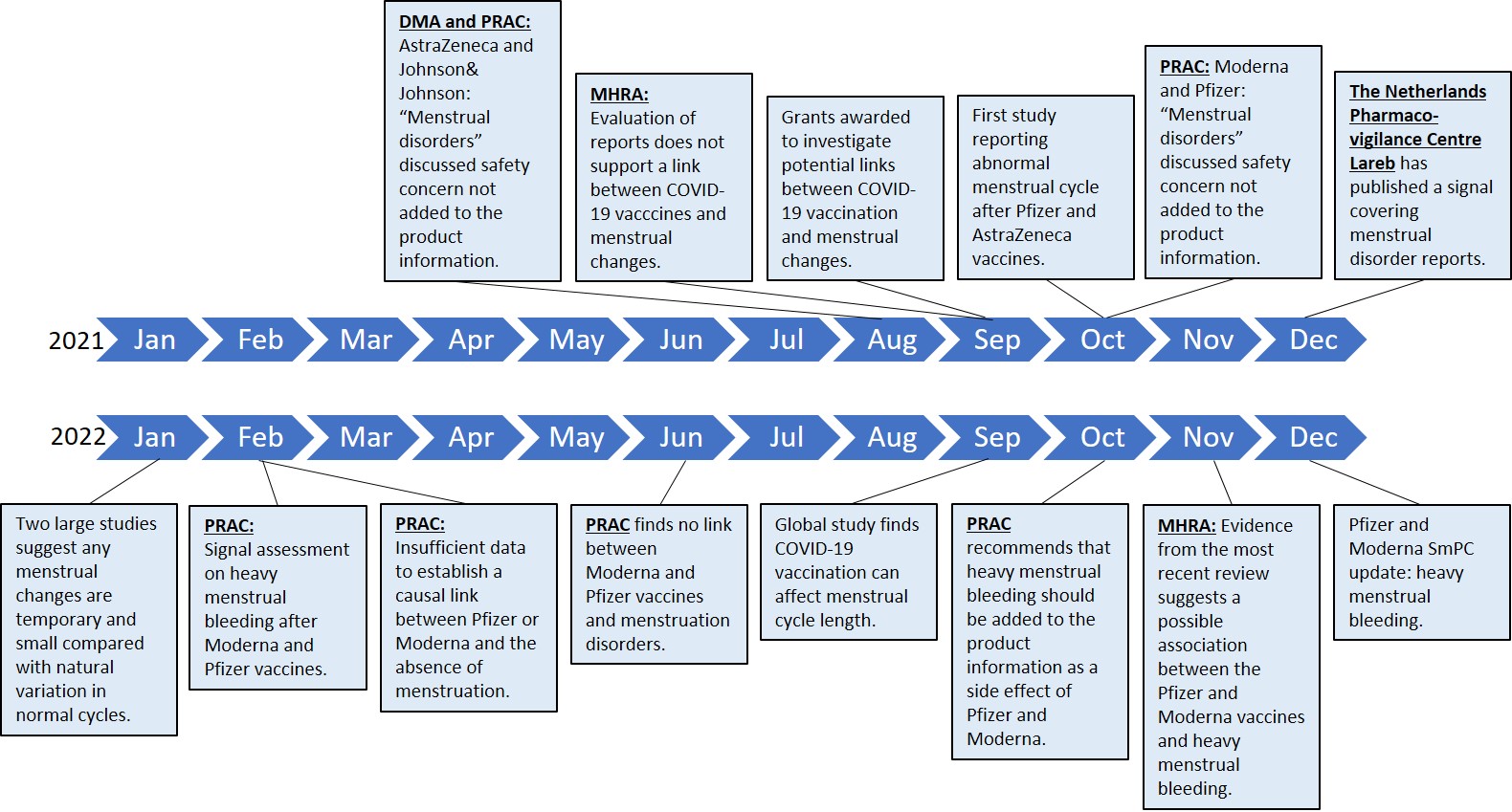
**

**Supplemental Figure 1.** Timeline of key events regarding menstrual disorders after the COVID-19 vaccines of Moderna, Pfizer, AstraZeneca, and Johnson&Johnson. DMA, Danish Medicines Agency; MHRA, Medicines and Healthcare products Regulatory Agency; PRAC, Pharmacovigilance Risk Assessment Committee

Supplemental Figure 2. Number of spontaneous reports of menstrual disorders after the COVID-19 vaccines of Moderna, Pfizer, AstraZeneca, and Johnson&Johnson over time, as received by The Netherlands Pharmacovigilance Centre Lareb.

## Supplemental File S3. Characteristics of included studies

| **First author** | **Publication year** | **Country** | **Study design** | **Vaccine type** | **Analyzed (n)** | **Age** | **Data source** | **Outcome(s)** | **Main finding(s)** | **Additional notes** |
| --- | --- | --- | --- | --- | --- | --- | --- | --- | --- | --- |
| Abdollahi [23] | 2022 | Iran | Cross-sectional | 46.6% Sinopharm, 26.6% AstraZeneca, 18.8% SputnikV, and 8% Covaxin | 427 Iranian female health workers | Mean 29.78 (SD 10.55) | Questionnaire | • Hypermenorrhea • Dysmenorrhea • Menorrhea | • 8% Sinopharm, 10.7% AstraZeneca, 5% SputnikV, and 17.6% Covaxin reported menstrual irregularities (hypermenorrhea-dysmenorrhea-menorrhea). • 8.8% menstruation disturbances and 4.6% metrorrhagia.  • Most commonly vaccine-induced change in menstruation was reported for Covaxin (17.6%), followed by AstraZeneca (10.7%), Sinopharm (8%) and SputnikV (5%).  • Differences between vaccines regarding menstruation disturbances were significant. |  |
| Akarsu [24] | 2022 | Turkey | Cross-sectional | 84.4% BioNtech-Pfizer, 15.1% Sinovac, 0.5% Turkovac | 590 undergaduate students | Mean 20.74 (SD 2.32) | One-to-one interview + questionnaire | • Menstrual irregularity | • 20.30% menstrual cycle irregularity after vaccination • 10.52% went to the doctor due to menstrual irregularity and received treatment | • Limitation: the study was conducted in one region and with young adults only, therefore the results cannot be generalized to the whole population. |
| Al-Furaydi [25] | 2023 | Saudi Arabia | Population-based cohort | Pfizer/BioNTech (first dose 83.8%, second dose 76.9%), Moderna (first dose 1.4%, second dose 6.9%) or Oxford/AstraZeneca (first dose 14.8%, second dose 16.2%) | 338 women | 18-45 years | Online survey | • Changes in menstrual cycle (shorter/longer interval between menstruations, missed period, less pain during menstruation, stronger pain during menstruation, more heavy bleeding than usual, less bleeding than usual, prolonged menstruation, short menstruation) | • 63.9% experienced variations in menstrual cycle after first or second dose.  • Highest rate in either the first (83.8%) or second (76.9%) dose after Pfizer/BioNTech.  • 51.1% menstrual cycle changes after first dose. | • Relatively minor alterations which usually resolve within two months. |
| Al-Mehaisen [26] | 2022 | Jordan | Cross-sectional | 46% Pfizer, 39% Sinopharm, 15% AstraZeneca | 1,506 women | 18-55 years (75% 26-45 years). | Online survey | • Changes in timing of the first period after the vaccine • Changes in duration of period • Changes in menstrual flow • Dysmenorrhea • New intermenstrual bleeding or postcoital bleeding | • 24.5% increased flow, 15.5% reduced flow (p-values 0.017). 23.6% delayed periods, 51.6% no changes. Changes in timing: p-value 0.008.  • Significant differences between the date of the last dose of vaccine with period timings, period duration, period volume, and dysmenorrhea after vaccine and period timings and period volume. • AstraZeneca was associated with new episodes or increased dysmenorrhea (P-value 0.035). |  |
| Alahmadi [27] | 2022 | Saudi Arabia | Retrospective cohort | Pfizer-BioNTech (first dose 75.8%, second dose 74.3%), Oxford-AstraZeneca (first dose 23%, second dose 21.8%), Moderna (first dose 1.2%, second dose 3.9%) | 673 females | 18-45 years | Questionnaire via social media platforms | • Menstrual cycle length • Amount of bleeding • Pain • Intermenstrual bleeding | • 46.7% changes in menstrual cycle after both doses, 22.9% more menstrual pain following the first dose, 21.4% after the second.  • Menstrual changes 65.2% in age groups 18-22 years, 65.4% in 38-45 years (65.4%) and 43.5% in 23-27 years, p<0.001. • Moderna highest rate of menstrual changes (65.4%), Oxford-AstraZeneca lowest rate (44.9%), p=0.040.  • The COVID-19 vaccination is associated with a minor and transient change in the menstrual cycle, resulting mainly more menstrual pain and increased bleeding. |  |
| Aldali [28] | 2022 | Saudi Arabia | Cross-sectional | Pfizer/BioNTech (first dose 98.1%, second dose 98.2%), Moderna (first dose 1.8%, second dose 6.5%) | 604 participants | 12-17 years (58.1% 16-17 years) | Questionnaire via social media platforms | • Menstrual irregularities | • Menstrual disorder: 123 (20.4%) after first dose, 106 (17.5%) after second dose • Patients who took the first dose and had a chronic disease had 2.4 times higher odds of having menstrual disorder (females) than non-chronic disease patients (p=0.03). | • Self-reported results of questionnaire.  • Recall bias may have occurred.  • Possible heterogeneity in participant responses because of subjective scale.  • The findings may not be applicable in a different area. |
| Alghamdi [10] | 2021 | Saudi Arabia | Cross-sectional | 62.3% Pfizer BioNTech, 37.6% Oxford-AstraZeneca | 4,170 participants | N.R. (Majority 20-30 years old) | Online survey | • Abnormal menstrual cycle (delaying/increase hemorrhages or pain) | • 18 cases (0.69%) irregular menstrual cycle after BNT162b2. 7 cases (0.45%) irregular menstrual cycle after ChAdOx1. | • Self-reported questionnaires, not clinically conﬁrmed by physicians. |
| Alvergne [29] | 2022 | United Kingdom | Prospective cohort + retrospective cohort | AstraZeneca (prospective 3.8%, retrospective 27.1%), Moderna (prospective 14.0%, retrospective 10.7%), Pfizer (prospective 82.3%, retrospective 61%), Janssen (prospective 0%, retrospective 0.6%) | 79 (prospective cohort), 1,273 (retrospective cohort) | 30 (median, prospective cohort), 33 (median, retrospective cohort) | Data collection tool and journal (prospective cohort), web-based form (retrospective cohort) | • Timing of menstruation (early or late)  • Blood flow | • COVID-19 vaccination is associated with a delay to the next period in spontaneously cycling individuals • Menstrual cycle returns to its pre-vaccination length in unvaccinated cycles • COVID-19 vaccination is not associated with any change to menstrual ﬂow • Brand of vaccine is not associated with differences in timing or ﬂow of next period • Timing of vaccination within the menstrual cycle does not have a clear effect on timing or ﬂow of next period | • Participants who noticed a change may be more motivated to return their journals.  • Lack of control group, no conclusions about causality can be drawn. |
| Alvergne [30] | 2023a | United Kingdom | Retrospective cross-sectional study | 53% Oxford-AstraZeneca, 47% Pﬁzer BioNTech | 4,989 pre-menopausal vaccinated participants  12,579 vaccinated vs. unvaccinated participants | Median 25 years, IQR 28-43. | Online survey | • Risk factors for reporting any menstrual changes following COVID-19 vaccination  • Menstrual frequency  • Regularity  • Duration  • Volume  • Inter-menstrual bleeding | • 18% menstrual cycle changes after ﬁrst COVID-19 vaccine  • Higher prevalence of menstrual changes for smoking, a history of COVID-19 disease, or not using estradiol-containing contraceptives.  • COVID-19 vaccination alone was not associated  with abnormal menstrual cycle parameters in the complete sample, while a history of COVID-19 disease was associated with an increased risk of reporting heavier bleeding, ‘‘missed’’ periods, and intermenstrual bleeding.  • No association between the brand of vaccine (Pﬁzer vs. AstraZeneca) nor the number of doses (1 vs. 2) with post-vaccination menstrual changes. |  |
| Alvergne [31] | 2023b | 110 countries  (49.7% United States, 14.1% United Kingdom, 5.0% Germany) | Retrospective cohort analysis | Pfizer-BioNTech, Oxford-AstraZeneca, CoronaVac/Sinovac,  Covishield, Johnson & Johnson/Janssen, Moderna, Sinopharm, and Sputnik V | 6,514 users from 110 countries (4,643 COVID-19 vaccination) | 16-45 years | Period tracker application Clue, linked to survey data on COVID-19 vaccination and disease  status. | • Menstrual cycle length changes | • The vaccinated group experienced a 1.14-day adjusted increase in cycle length during cycle 4 (COVID-19 vaccine) compared with their preevent average (95% CI 0.60-1.69). Changes resolve quickly within the next cycle.  Experiencing COVID-19 was associated with a small and temporary change in cycle length similar to that with  COVID-19 vaccination. |  |
| Amer [32] | 2022 | Saudi Arabia, Egypt, Syria, Libya, and Sudan | Cross-sectional | 46.1% Pfizer, 24.3% Oxford-AstraZeneca, 8.8% Sinopharm, 3.2% Septotic light, 3.1% Senophak, 2.2% Jonson and Jonson, 0.9% Moderna | 1,044 vaccinated Arab women between 15-50 years | Mean 30.4 (SD 3.9) | Questionnaire | • Menstrual changes (blood loss, period duration, pelvic and abdominal pain, back pain, breast pain, mood changes, headache, fatigue, ovulatory pain, dyspareunia) | • The 1,044 (83.5%) vaccinated females reported 418 (38.5%) MCs after being vaccinated, and these MCs resolved in 194 women (55.1%) after more than 9 months.  • MCs were reported at 293 (80.6) after the 2nd dose, and were mainly reported after 482 (46.1) Pﬁzer, 254 (24.3) AstraZeneca, and 92 (8.8) Sinopharm. |  |
| Anjorin [33] | 2022 | Africa | Descriptive, cross-sectional | 77.8% Oxford-AstraZeneca, 9.1% Pfizer-BioNTech, 4.5% Sinopharm-BBIBP, 1.8% Moderna, 0.9% Covaxin, 0.7% Sputnik V, 0.6% CoronaVac, 0.5% Johnson&Johnson, 0.5% Sinopharm-WIBP, 0.2% Covi Vac, 3.3% other | 969 Africans from 35 countries | N.R. (18 - >65 years) | Online survey | • Menstrual disorder | • Menstrual disorder 0.5% (n = 5) |  |
| Baena-García [34] | 2022 | Spain | Cross-sectional retrospective study, part of The Effect of Vaccination against SARS-CoV-2 on the Menstrual Cycle (EVA Project)” | 61.1% Pfizer-BioNtech, 16.3% Oxford/AstraZeneca, 17.4% Moderna, 5.3% Johnson&Johnson/Janssen | 14,153 women | Mean 31.5 (SD 9.3) | Online survey | • Amount and duration of menstrual bleeding • Presence of clots • Cycle length • Premenstrual symptoms | • 78% menstrual cycle changes after vaccination. • Women who reported menstrual changes after vaccination were older (overall p < 0.001) and slightly more smokers (p = 0.05) than women who did not report any changes.  • The most prevalent changes in relation to premenstrual symptoms were increased fatigue (43%), abdominal bloating (37%), irritability (29%), sadness (28%), and headaches (28%).  • The most predominant menstrual changes were more menstrual bleeding (43%), more menstrual pain (41%), delayed menstruation (38%), fewer days of menstrual bleeding (34.5%), and shorter cycle length (32%). • Other results: increased need for medication (32%%), more or larger clots (29%), less menstrual bleeding (23%), more days of menstrual bleeding (21%), fewer or smaller clots (16%), less menstrual pain (10%), decreased need for medication (6%) • Women vaccinated with Pfizer or Moderna (ARN-mdesign/technology based) reported less premenstrual and menstrual-related symptomatology than those vaccinated with Astra-Zeneca or Janssen (adenovirus vectored design/technology based) (overall p = 0.004). |  |
| Barabás [35] | 2022 | Hungary | Cross-sectional, retrospective analysis | 56.3% Pfizer-BioNTech, 23.8% AstraZeneca, 12.4% Sputnik, 8.4% Sinopharm, 5.9% Moderna, 1.2% Janssen | 1,563 women | 18-65 years | Online survey | • Cycle length • Menses length • Cycle regularity | • Menstrual cycle length did not change in any of the periods.  • Menses length increased, regularity of the menstrual cycle decreased signiﬁcantly during the peak of the COVID-19 pandemic when comparing to the pre- and post-peak periods.  • Menstrual cycle abnormalities during the peak of COVID-19 in Hungary might be the result of elevated depressive symptoms. • 40.4% menstrual cycle disturbances after receiving COVID19 vaccines. • Irregular bleeding (12.2 %), heavier bleeding (4.3 %), strong menstrual cramps (2.8 %) and period reappearance (2 %). • 43.4 % of the participants receiving vaccine experienced menstrual problems after the ﬁrst, while 41.3 % after the second shot of vaccine. 12.5 % reported menstrual cycle changes after both doses, 1.6 % encountered menstrual problems after the third vaccination only, and 1.2 % after different combinations of the vaccinations. | • The collected data may contain some bias such as social acceptance error.  • Recall bias may also be a problem since the study was self-reporting and asked questions for an interval of more than one year. Furthermore, the overrepresentation of highly qualiﬁed individuals may also lead to bias as women’s reproductive health is highly inﬂuenced by their socioeconomic status. |
| Bisgaard Jensen [36] | 2023 | Denmark | Population-based cohort study, as part of the BiCoVac Cohort | 78% Pfizer-BioNtech, 20% Moderna, <1% other | 13,648 vaccinated, menstruating women | 16-65 years | Survey | • Any self-reported menstrual change (menstrual cycle length, heavier/lighter bleeding, more (ir)regular menstrual cycles, menstrual absence, intermenstrual bleeding, prolonged bleeding, shortened bleeding, two monthly menstrual bleedings)  • Potential risk factors of menstrual changes | • 30% menstrual changes following COVID-19 vaccination.  • 18% [16.61–19.34] change duration of more than 4 months.  • Change in menstrual cycle length was the most frequently reported change, (9% [8.24–9.20] reported longer menstrual cycles and 7% [6.60–7.47]  reported shorter menstrual cycles).  • Heavier bleeding 7% [6.31–7.15], more irregular menstrual cycles 7% [6.85–7.72]. 8% [7.93–8.87] ‘changed in other ways’ of which menstrual absence, intermenstrual bleeding, and prolonged bleeding were the most  reported changes.  • Most of the potential risk factors were associated with reports of menstrual changes following COVID-19  vaccination. In particular, higher odds were found among women who reported 5 immediate vaccine symptoms; OR 1.67[1.50–1.86], had had a prior severe COVID-19 infection; OR 2.17 [1.40–3.35], had a high-stress level at baseline; OR 1.67 [1.32–2.10],  or were concerned about COVID-19 vaccines prior to vaccination; OR 1.92 [1.50–2.45]. Lower odds were found among women with  regular menstrual cycles using hormonal contraception; OR 0.71 [0.65–0.78]. | Unable to address the causal effect of COVID-19 vaccination on the reported  menstrual changes, as information about menstrual changes was not available among non-vaccinated women. |
| Caspersen [37] | 2023 | Norway | Self-controlled case series, as part of the Norwegian Mother, Father and Child Cohort Study (MoBa) | 99.9% Comirnaty, 0.1% Vaxzevria or Spikevax | 1,468 girls who had started to menstruate | 12-15 years | Online survey | • Heavier bleeding than usual • Prolonged menstruation • Shorter interval between menstruations than usual • Longer interval between menstruations than usual • Spot bleedings between menstruations • Stronger pain during menstruation • Period pain without bleeding • Any other symptoms from the pelvic region | • 25.1% at least one event for the first cycle after vaccination. • Of these, one single menstrual irregularity was reported by 14.2%, two different irregularities were reported by 6.1%, three irregularities by 2.8%, and four or more irregularities by 2.0%. • Vaccinated girls had more than twofold increased risk of reporting heavy menstrual bleeding and prolonged bleeding after vaccination compared to the last cycle for unvaccinated subjects.  • The risk of heavier menstrual bleeding and prolonged bleeding was higher in the menstrual cycle after vaccination than in the cycle before vaccination, RRs 1.61 (95%CI 1.43-1.81) and 1.40 (95%CI 1.23-1.60), respectively.  • Vaccination was also associated with increased risk of shorter interval, longer interval, and stronger period pains (RRs 1.14 to 1.19).  • No association between vaccination and spot bleeding, period pains without bleeding, or other symptoms from the pelvic region. | • Mothers filled out the questionnaire.  • Questionnaires regarding menstrual disorders pre- and post vaccination were filled out at the same moment. |
| Cheng [38] | 2022 | China | Cross-sectional | Sinopharm. CoronaVac. 1047 (75%) two doses | 1392 healthcare workers in perinatal medicine and obstetrics/gynecology aged <=50 years (1264 (90.8%) female) | Range 18-50 years | Survey | • Menstrual delay • Early menstruation • Menorrhagia • Prolonged period | • Menstrual delay (1.4% first dose, 0.6% second dose) • Early menstruation (0.4% first dose, 0.3% second dose) • Menorrhagia (0.2% first dose, 0.1% second dose) • Prolonged period (0.1% first dose, 0% second dose) | • Whether the irregular menstrual change was associated with COVID-19 vaccination or was a co-incident event requires further investigation. |
| Chiang [39] | 2022 | Taiwan | Cross-sectional | Oxford AstraZeneca (first dose 65%, second dose 60%), Moderna or Pfizer-BioNTech (first dose 35%, second dose 40%) | 20 female athletes | Mean: 21 | Questionnaires | • Menstruation duration (days) • Menstrual cycle (days) | • No significant changes in the menstruation cycle or days of the menstrual period (p>0.05). |  |
| Dabbousi [40] | 2022 | Lebanon | Cross-sectional | 81.2% Pfizer, 11.9% AstraZeneca, 4% Sputnik, 2% Sinopharm, 0.2% Moderna | 505 Lebanese adult women | Mean 26.9 years (range 18-55 years) | Online survey | • Heavy bleeding • Light bleeding • Pain • Irregularity in cycle duration • Duration • Regularity of cycle | • Around two-thirds no change in menstruation after taking the vaccine.  • Heavy bleeding decreased (39% vs. 33%, p=0.003), light bleeding increased (27% vs. 34%, p<0.001). • Regular cycles decreased after taking the vaccine (88% vs. 80%, p < 0.001). • Women using hormonal contraception method or using any hormonal therapy had higher menstrual irregularity rates (p = 0.002 and p = 0.043, respectively). |  |
| Dar-Odeh [41] | 2022 | Jordan and Saudi Arabia | Cross-sectional | 48.4% Pfizer-BioNTech, 31.7% Sinopharm, 18.3% AstraZeneca | 498 physicians and dentists (70% female) | Mean 35.75 (SD 11.74), range 22-71 years | Questionnaire | • Menstrual disturbances | • Menstrual disturbances: n = 15 (4.8%) out of 314 menstruating women |  |
| Darney [42] | 2023 | United Kingdom (32.4%), Europe (31.2%), U.S.A. and Canada (30.1%), Australia and New Zealand (3.9%), Other (2.4%) | Retrospective cohort study | 66.7% Pfizer-BioNTech, 17.9% Moderna, 8.8% Astrazeneca, 2.0% Johnson&Johnson | 9,555 individuals (7,401 vaccinated and 2,154 unvaccinated) | 18-45 years | App Natural Cycles | • The mean number of heavy bleeding days (fewer, no  change or more)  • Changes in bleeding quantity (less, no change or more) at three time points (first dose, second dose and post- exposure menses). | • About two- thirds of individuals no change in the number of heavy bleeding days, regardless of vaccination status. After adjusting for confounding factors, no significant differences in the number of heavy bleeding days by vaccination status.  • Increase in total bleeding quantity (34.5% unvaccinated, 38.4% vaccinated; adjusted difference 4.0%, 99.2% CI 0.7%– 7.2%).  • Differences resolved in the cycle post- exposure.  • Small increase in the probability of greater total bleeding quantity occurred following the first COVID- 19 vaccine dose, which resolved in the cycle after the post- vaccination cycle. The total number of heavy bleeding days did not differ by vaccination status.  • Findings can reassure the public that any changes are small and transient.  • No major differences in change in number of heavy bleeding days or total bleeding quantity when comparing individuals who received an mRNA vaccine with individuals who received an adenovirus vector vaccine during either the first- or second- dose menses,  and no differences in any bleeding outcome between the two mRNA vaccines (data not shown).  • The number of individuals who received an inactivated virus (n = 44 for first- dose menses and n = 31 for second- dose menses) was too small to draw conclusions. No meaningful differences were found in the outcomes when examined by timing of vaccination (during menses vs after completion of menses). | • Large global sample with geographic diversity and prospectively tracked bleeding data. |
| Dellino [43] | 2022 | Italy | Retrospective, descriptive study | 43% Pfizer/BioNTech (Comirnaty), 32% Moderna, 25% Astrazeneca (Vaxzervria) | 100 women | Mean age 33 years, range 18-45 | Questionnaire | • Delayed menstruation • Abnormal uterine bleeding | • Symptoms such as delayed menstruation and abnormal uterine bleeding (metrorrhagia, menometrorrhagia, and menorrhagia) were generally reported within the ﬁrst three weeks of vaccination, especially after the second dose, with a percentage of 23% and 77%, respectively.  • 77% had abnormal uterine bleeding (AUB), of which 47% had metrorrhagia, 30% had menometrorrhagia, and 23% had menorrhagia | • The period of observation and the size of the sample should be extended with a multicenter trial in order to provide a greater number of reports and evidence and implement the acquisitions of the AIFA. |
| Duijster [17] | 2023 | The Netherlands | Prospective cohort study | Spontaneous reports: 79% Pfizer, 11% Moderna, 7% Johnson&Johnson, 3% AstraZeneca | 16,929 women in cohort study +  24,090 spontaneous reports | Spontaneous reports: <25 - >=65 years. 38% 25-34 years.  Cohort: <25-64 years. 56% 25-34 years. | Spontaneous reporting system/web-based reporting form and prospective cohort event monitoring | • Amenorrhoea/oligomenorrhoea  • Dysmenorrhoea  • Heavy menstrual bleeding  • Intermenstrual blood loss  • Irregular blood loss  • Less menstrual blood loss  • Abnormal withdrawal blood loss  • Other | • Amenorrhoea/oligomenorrhoea (33%)  • Dysmenorrhoea (13%)  • Heavy menstrual bleeding (29%)  • Intermenstrual blood loss (19%)  • Irregular blood loss (23%)  • Less menstrual blood loss (4%)  • Abnormal withdrawal blood loss (1%)  • Other (14%)  • The CEM study showed an incidence of 41.4 per 1000 women aged ≤54 years.  • Significant associations observed for 25–34 years (odds ratio 2.18; 95% confidence interval 1.45–3.41) and the Pfizer vaccine (odds ratio 3.04; 95% confidence interval 2.36–3.93).  • No association for body mass index and presence of most comorbidities assessed.  • The use of hormonal contraceptives was not consistently associated with the occurrence of menstrual  abnormalities. |  |
| Edelman [13] | 2022a | United States of America | Retrospective cohort study of prospectively collected data; vaccinated vs. unvaccinated | 55% Pfizer/BioNTech, 35% Moderna, 7% Johson & Johnson/Janssen, 3% unspecified | 3,959 individuals (2,403 vaccinated; 1,556 unvaccinated) | 18-45 years, majority 25-29 years (37%) | Application "Natural Cycles" | • Cycle length • Menses length | • COVID-19 vaccine was associated with a less than 1-day change in cycle length for both vaccine-dose cycles compared with prevaccine cycles (first dose 0.71 day-increase, 98.75% CI 0.47–0.94; second dose 0.91, 98.75% CI 0.63–1.19) • Unvaccinated individuals saw no significant change compared with three baseline cycles (cycle four 0.07, 98.75% CI 20.22 to 0.35; cycle five 0.12, 98.75% CI 20.15 to 0.39). In adjusted models, the difference in change in cycle length between the vaccinated and unvaccinated cohorts was less than 1 day for both doses (difference in change: first dose 0.64 days, 98.75% CI 0.27–1.01; second dose 0.79 days, 98.75% CI 0.40–1.18).  • Change in menses length was not associated with vaccination. | • Strengths: prospectively collected menstrual cycle data, which limits recall bias, a control group of unvaccinated individuals, large sample size, and adjustment for sociodemographic factors associated with vaccination status and menstrual cycle changes (eg, age, BMI).  • Limitation: the study only assessed two aspects of the menstrual cycle. |
| Edelman [44] | 2022b | United Kingdom (32%), United States of America and Canada (29%), Europe (33%), Australia (4%), other (2%) | Retrospective cohort study of prospectively collected data; vaccinated vs. Unvaccinated | 66% Pfizer-BioNTech, 17% Moderna, 9% Oxford-AstraZeneca, 1.9% Johnson % Johnson, 0.5% other, 4.5% unspecified | 19,622 individuals (14,936 vaccinated, 4,686 unvaccinated) | 18-45 years (80% <35 years), mean 30 years | Application "Natural Cycles" | • Cycle length • Menses length | • Individuals who were vaccinated had a less than one day adjusted increase in the length of their first and second vaccine cycles, compared with individuals who were not vaccinated (0.71 day increase (99.3% confidence interval 0.47 to 0.96) for first dose; 0.56 day increase (0.28 to 0.84) for second dose). The adjusted difference was larger in people who received two doses in a cycle (3.70 days increase (2.98 to 4.42)). One cycle after vaccination, cycle length was similar to before the vaccine in individuals who received one dose per cycle (0.02 day change (99.3% confidence interval −0.10 to 0.14), but not yet for individuals who received two doses per cycle (0.85 day change (99.3% confidence interval 0.24 to 1.46)) compared with unvaccinated individuals. Changes in cycle length did not differ by the vaccine’s mechanism of action (mRNA, adenovirus vector, or inactivated virus).  • Changes were small and resolved as soon as the next cycle after vaccine receipt, except in people who received both doses in one menstrual cycle. • Menses length was unaffected by vaccination. | • This study was performed after the U.S. cohort study (Edelman 2022a). This study represents a larger, more geographically diverse population receiving a broader range of vaccine types and brands as well as differing vaccine timing schemes than their previous publication. • Strengths: robust study design and analytical methodology, the inclusion of an unvaccinated comparison group, and prospectively tracked menstrual cycle data. These items altogether mean that the outcomes are not affected by recall bias, either due to cross-sectional documentation of vaccine dosage and outcomes or the natural variation in menstrual outcomes. |
| El-Shitany [45] | 2022 | Saudi Arabia | Retrospective cross-sectional | 100% Pfizer BioNTech (2nd and/or 3rn doses only) | 442 people, all of Arab heritage (72% female) | 83% <=60 years | Online survey | • Dysmenorrhoea and heavy menstruation | • Dysmenorrhoea and heavy menstruation (dose 2 0.6%, dose 3 0.6%, Chi square p=1.000). | • The current study is one of the leading studies investigating the adverse reactions of Pfizer BioNTech COVID-19 vaccine booster dose. |
| Farah [46] | 2023 | Lebanon | Cross-sectional | 99% Pfizer-BioNTech | 304 female Lebanese health care workers who were vaccinated and aged 18 to 65 years (240 premenopausal and 64 postmenopausal) | 18-65 years | Survey | • Change in cycle length  • Change in cycle flow  • Breakthrough bleeding  • Duration of menstruation  • Change in symptoms  • Vaginal bleeding among postmenopausal women | • Change in cycle length: 6.9% after first dose, 9.2% after second dose, 6.9% after third dose.  • Change in menstrual flow: 5.6% after first dose, 7.2% after second dose, 6.9% after third dose.  • Breakthrough bleeding: 1% after each dose.  • Symptoms: 77%  • Duration: 3-3.5% after each dose.  • Vaginal bleeding: 2%  • Change in cycle length was significantly associated with age (P = 0.025 after the first dose and P = 0.017 after the second dose), level of education (P = 0.013 after the first dose and P = 0.012 after the second dose), and fibroids (P = 0.006 after the second dose and P = 0.003 after the third dose). • Change in cycle flow was significantly associated with age (P = 0.028), fibroids (P = 0.002 after the second dose and  P = 0.002 after the third dose), bleeding disorders (P = 0.000), and chronic medications (P = 0.007).  • Change in symptoms was associated with polycystic ovary syndrome (P = 0.021), chronic medications (P = 0.019 after the second dose and P = 0.045 after the third dose), and fibroids (P = 0.000). |  |
| Farhat [47] | 2022 | Saudi Arabia | Cross-sectional retrospective study | 1st dose 78% Pfizer, 22% Ofxord. 2nd dose 83% Pfizer, 17$ Oxford. 3rd dose 99% Pfizer, 1% Oxford. | 1018 individuals (62% female) | 12 to >65 years. 42% 30-49 years. | Online survey via social media | • Menstrual changes (mainly irregular cycles, increased length of menstrual cycle, and increased menstrual flow) | • 18 females (1.82%) experienced menstrual changes after receiving the first vaccine dose, of these 15 females received Pfizer vaccine and 3 females received the Oxford vaccine.  • After the second dose of the Pfizer vaccine, 15 females (1.53%) reported menstrual disturbances. These changes were mainly irregular cycles, increased length of menstrual cycle, and increased menstrual flow. | • These changes cannot be directly attributed to the COVID-19 vaccines because of the relatively low number of reported menstrual changes following COVID-19 vaccine administration compared to the total number of vaccines given and the prevalence of menstrual disorders in general. Since menstrual disturbances are common and can be influenced by many factors, further research is needed to study its possible association with COVID-19 vaccines. |
| Farland [48] | 2022 | United States of America | Longitudinal study | 63% Pfizer-BioNTech, 33% Moderna, 4% Janssen | 545 women of the Arizona COVID-19 Cohort (CoVHORT) study | Mean 32.5 years (SD 6.4), range 18-45 years. | Three questionnaires (baseline, 3 months, 6 months) | • One or more missed menstrual periods • Infrequent menstruation • Irregular menstruation • Abnormal bleeding or spotting between normal menstrual periods • Abnormal heavy or prolonged bleeding • Abnormal light bleeding • Increase in menstrual pain or cramps • Increase in premenstrual symptoms | • Approximately 25% of vaccinated participants reported a change in their menstrual cycle after vaccination; the majority reported changes after their second dose (56%) as compared with their ﬁrst (18%) and third (14%) doses.  • Irregular menstruation (43%), increased premenstrual symptoms (34%), increased menstrual pain or cramps (30%), and abnormally heavy or prolonged bleeding (31%). High self-reported perceived stress levels compared with low perceived stress (OR, 2.22; 95% CI 1.12-4.37) and greater body mass index (OR, 1.04; 95% CI 1.00-1.07) were associated with greater odds of experiencing the menstrual cycle changes after the vaccination.  • Participants having a history of SARS-CoV-2 infection were less likely to report changes in their menstrual cycle after vaccination compared with the participants with no history of SARS-CoV-2 infection (OR, 0.58; 95% CI 0.32-1.04). | • Among vaccinated participants, approximately 25% of them reported predominantly temporary changes in the menstrual cycle, however, they were unable to determine whether these changes are due to normal cycle variability. |
| Filfilan [49] | 2023 | Saudi Arabia | Cross-sectional | 64% Pfizer-BioNTech, 16% Sinovac, 8% AstraZeneca/Oxford, 8% Sinopharm, 3% Moderna, 1% Sputnik | 2,381 vaccinated females aged 15-49 years.  Females from the Kingdom of Saudi Arabia (70%), Pakistan (24%), Bahrain, United Arab Emirates, Kuwait, Iraq, Egypt, Dubai, Sri Lanka, Jordan, Algeria, Australia, Serbia, and Tunisia took part in the survey. | Mean 25±7.7 years | Online survey | • Change in cycle length  • Change in flow of menstrual bleeding  • Dysmenorrhea  • Changes in intensity  • Intermenstrual bleeding | • 67% observed post-vaccination menstrual changes.  • Approximately 357 (22%) study participants reported changes after the first dose, 876 (55%) after the second dose, and 13 (1%) females after the booster, and these findings were statistically significant (p<0.001).  • A strong association (p=.008) was found between the type of vaccine and changes in the menstrual cycle in  participants (AstraZeneca 11 (36%)) after one dose. A strong association (p=.004) was also seen between the type of vaccine (Pfizer 543 (83%)) and menstrual changes after the booster dose. Cycles became irregular 180 (36%) or prolonged 144 (29%) in females inoculated with Pfizer after two doses of vaccination (p=0.012).  56% of changes were related to the duration of the menstrual cycles, which became either irregular (unscheduled bleeding) in 596 (35%) cases, prolonged (longer than 35 days) in 476 (28%) cases, or short in 311 (18%) cases. |  |
| Gibson [50] | 2022 | United States of America | Prospective cohort study (Apple Women's Health Study (AWHS)) | 55% Pfizer-BioNTech, 37% Moderna, 7% Johnson & Johnson/Janssen | 9,652 participants (8,485 vaccinated, 1,166 unvaccinated) | Range <20 to >=50 | Online survey + cycle tracking app | • Menstrual cycle length | • Among vaccinated participants, COVID-19 vaccination was associated with a small increase in mean cycle  length (MCL) for cycles in which participants received the first dose (0.50 days, 95% CI: 0.22, 0.78) and cycles in which participants received the second dose (0.39 days, 95% CI: 0.11, 0.67) of mRNA vaccines compared with pre-vaccination cycles. Cycles in which the single dose of J&J was administered were, on  average, 1.26 days longer (95% CI: 0.45, 2.07) than pre-vaccination cycles. Post-vaccination cycles returned to average pre-vaccination length. Estimates for pre vs post cycle lengths were 0.14 days (95% CI: -0.13, 0.40) in the first cycle following vaccination, 0.13 days (95% CI: -0.14, 0.40) in the second, -0.17 days (95% CI: -0.43, 0.10) in the third, and -0.25 days (95% CI: -0.52, 0.01) in the fourth cycle post-vaccination. • When a second dose was received in the luteal phase, that cycle was, on average, shorter. Doses received in the follicular phase explained the increase in mean cycle length observed in the primary results. | • Menstrual cycle change following COVID-19 vaccination appears small and temporary.  • Strengths: large sample size allowed for sufficient statistical power to detect minor differences measured in fractions of days. More than one app used. |
| Hariton [51] | 2023 | United States of America | Cross-sectional | Not reported | 5,314 participants aged 18-55 years | 18-55 years | Menstrual tracker app “Glow” | Change in cycle length | • Neither receipt of the COVID-19 vaccine nor recent SARS-CoV-2 infection led to meaningful aberrations in menstrual cyclicity. |  |
| Issakov [52] | 2022 | Israel | Cross-sectional | 100% Pfizer | 7,476 COVID-19 vaccinated women in Isreal | > 18 years | Online survey via social media | • Menstrual changes including amount, duration, and frequency | • 49.3% experienced abnormal uterine bleeding after being vaccinated, of which 81% excessive.  • Women who reported excessive bleeding were characterized by a history of extended and heavy menses, a greater use of non-hormonal IUDs, and by having been vaccinated according to protocol at higher rates compared to those who experienced no or scant bleeding. |  |
| Kajiwara [53] | 2023 | Japan | Cross-sectional | 91% Moderna, 9% Pfizer | 55 female Japanese students | 18-22 years approximately | Survey | • Menstrual cycle length | • The difference between the predicted and actual menstrual cycle length was 1.9 ± 3.0, 1.6 ± 2.8 (p = 0.557), and 2.5 ± 3.8 (p = 0.219) days before vaccination and after the first and second dose of the vaccine, respectively.  • In participants who received vaccinations twice within a single menstrual cycle, this difference was 1.3 ± 3.5 and 3.9 ± 3.3 (p = 0.045) days before and after vaccination, respectively. • The grade and proportion of side effects that occurred after the second dose of the COVID-19 vaccine tended to be the highest during the menstrual period and the lowest during the ovulation period. | • Small sample size and targeted female students at a medical university, thus it was a limited group of Japanese adults. |
| Laganà [54] | 2022 | Italy | Cross-sectional | 5% Vaxzevria (AstraZeneza), 81% Comirnaty (Pfizer-BioNTech), 12% Spikevax (Moderna), 2% Janssen (Johnson & Johnson) | 164 women | 35.8 (SD 7.2) years | Online survey | • Alterations in frequency of the subsequent menstrual cycle • Alterations in the length of the subsequent menstrual cycle • Alterations in the quantity of the subsequent menstrual flow | • 50–60% menstrual cycle irregularities after first dose , regardless of the type of administered vaccine. 60-70% after second dose. Menstrual irregularities after both the ﬁrst and second doses of the vaccine were found to self-resolve in approximately half the cases within two months. |  |
| Lee [55] | 2022 | United States of America | Cross-sectional | 55% Pfizer, 33% Moderna, 2% AstraZeneca, 9% Johnson & Johnson (excluded), 3% Other | 39,129 current and formerly menstruating adults (91% women-only identifying and 9% gender-diverse) | 18-80 years, mean 34.22 years (SD 9.18) | Online survey | • Menstrual bleeding changes (by regularly menstruating respondents) • Breakthrough bleedings (by nonmenstruating respondents) | • 42% of people with regular menstrual cycles bled more heavily than usual, while 44% reported no change after being vaccinated. • Among respondents who typically do not menstruate, 71% of people on long-acting reversible contraceptives, 39% of people on gender-affirming hormones, and 66% of postmenopausal people reported breakthrough bleeding.  • Increased/breakthrough bleeding was significantly associated with age, systemic vaccine side effects (fever and/or fatigue), history of pregnancy or birth, and ethnicity. | • This is the very first characterization of postvaccine menstrual bleeding changes for a gender-diverse sample of pre- and postmenopausal people.  • The associations reported here cannot establish causality. • Self-reporting and no control group |
| Lessans [56] | 2022 | Israel | Cross-sectional | 100% Pfizer/BioNTech | 219 women | Mean 29.6 years (SD 8.6). Range 18-50 years. | Online survey | • Irregular bleeding • Presence of any menstrual change, including irregular bleeding, mood changes, or dysmenorrhea | • 51 (23.3%) irregular bleeding. Of them, 20 (39. 2%) and 31 (60.8%) reported irregular bleeding after the first and second doses of the SARS- CoV- 2 BNT162b2 mRNA vaccine, respectively. 34 (66.7%) of them reported irregular bleeding that preceded their estimated menstrual date (mean 9.9 ± 3.0 days) and 17 (33.3%) reported a delay in their expected menstrual date (mean 12.3 ± 6.3 days).  • 37% (n = 83) any menstrual change following vaccination.  • 68% dysmenorrhea.  • 56% other menstrual symptoms (abdominal pain, pelvic pain, appearance of new acne, breast tenderness, hot flushes).  • 9.6% mood changes associated with menstruation.  • Parity was positively associated with irregular bleeding with 26 (50%) of those suffering from irregular bleeding being multiparous compared with only 53 (31.5%) of women with no irregular bleeding (nulliparous 46% vs 60%, multiparous 50% vs 31%, rest 4% vs 8%, P = 0.049). The presence of medical comorbidities was also significantly higher among patients who experienced irregular bleeding (20.0% vs 6.0%, P = 0.003). |  |
| Ljung [57] | 2023 | Sweden | Cohort study | Pfizer-BioNTech, Moderna, AstraZeneca | 2,946,448 Swedish women aged 12-74 years, of whom 2,580,007 (87.6%) vaccinated | Median 46 (25-59 years) of women who received at least one dose | Data from Swedish national and regional registers | • Postmenopausal bleeding  • Menstrual disturbance  • Premenopausal bleeding | • The highest risks for bleeding in women who were postmenopausal were observed after the third dose, in the one to seven days risk window (hazard ratio 1.28 (95% confidence interval 1.01 to 1.62)) and in the 8-90 days risk window (1.25 (1.04 to 1.50)). Risk of postmenopausal bleeding suggested a 23-33%  increased risk after 8-90 days with BNT162b2 and mRNA-1273 after the third dose, but the association with ChAdOx1 nCoV-19 was less clear. For menstrual disturbance or bleeding in women who were premenopausal, adjustment for covariates almost completely removed the weak associations noted in the crude analyses.  These findings do not provide substantial support for a causal association between SARS-CoV-2 vaccination and healthcare contacts related to menstrual or bleeding disorders. | • Strengths: populationbased  cohort design, large  sample size, near complete  follow-up, and independent ascertainment  of data for SARS-CoV-2  vaccinations and healthcare contacts from nationwide registers with mandatory reporting,  in a setting with a universal, tax financed  healthcare system. |
| Matar [58] | 2022 | Jordan, Palestine, Syria, Egypt, Sudan, and Libya | Cross-sectional | 27.2% Pfizer, 24.7% Sinopharm or Sinovac, 24.3% AstraZeneca, 17.4% Sputnik, 4.6% Johnson and Johnson, 1.7% Moderna | 4942 menstruating females (2919 (59%) vaccinated) | Mean 24.04 years (SD 5.73) | Online survey | • Menstrual changes including a variety of symptoms (menstrual flow, menstrual cycle length, pain) | • COVID-19 vaccine was significantly associated with increased back pain (83% vs. 78%, p>0.001), nausea (43% vs. 40%, p=0.036), tiredness, pelvic pain with periods (86% vs. 82%, p=0.006), administration of over the counter analgesics (62% vs. 57%, p>0.001), bowel movement (p=0.002), looseness of the stool (p=0.012), and pain score on average (p>0.001). An increase in the heaviness of bleeding was reported among females who were fully vaccinated. • By comparing the six vaccine types, a higher percentage of menstrual irregularity was observed in Johnson & Johnson, followed by Sinopharm, Moderna, and AstraZeneca (p = 0.022). Similarly, Johnson & Johnson was associated with a higher percentage of heavy bleeding with coagulations, followed by Pfizer, Sinopharm, AstraZeneca, and Moderna (p = 0.003). |  |
| Minguez-Esteban [59] | 2022 | Spain | Cross-sectional | 70% Pfizer, 30% Moderna in the ﬁrst dose, 69% and 31% in the second dose. | 746 Spanish women | 63% between 18-30 years, 37% between 31-45 years | Online survey | • Menstrual pain • Menstrual cycle length • Amount of bleeding | • Sixty-ﬁve per cent of the women perceived changes in their menstrual cycle after receiving the vaccines, irrespective of the type of vaccine or number of doses. • All p values were >0.05. Older participants more frequently reported alterations (77% of women between 31–45 years) than younger ones (60% of woman between 18–30, ­X2 (1) = 11.18, Z = 3.3, p < 0.001. • Unlike pain and bleeding, women between 31–45 years old experienced a greater rise in duration of MC (0.44 ± 1.26 days) than younger women (0.09 ± 1.26 days), Z = 3.68, p < 0.001. • Longer duration of menstrual cycle (24%), shorter duration of menstrual cycle (12%), higher bleeding amount (17%), lower bleeding amount (9%), more pain (24%), less pain (3%), absence of menstrual cycle (10%) | • The novelty of the present study is that it conﬁrms that menstrual cycle alterations in this sample could last more than 5 months after vaccination. |
| Mohr-Sasson [60] | 2023 | Israel | Cohort study | 100% Pfizer-BioNTech | 35 adolescent girls aged 12-16 years who were vaccinated by two Pfizer-BioNTech Covid-19 vaccines | Mean 13.8 (± SD 1.26) | Computerized questionnaire | • Change in menstrual regularity  • Changes in menstrual intensity  • Changes in menstrual  length  • Estimated change in anti-Mullerian Hormone (AMH) levels | • Among the 22/35 girls who reported regular menstruation before vaccination, seven (31.8%) experienced irregularities post-vaccination.  • On follow-up 3 months after receiving the first vaccine, 4/8 (50%) of the pre-menarche girls reported experiencing  their first menstruation. Among the girls reporting regular menstruation (n = 23), two (8.9%) suffered from irregularity during the first month following  vaccination that has resolved, and in five (21.7%) girls the pattern has continued to be  irregular on the 3 month  follow-up visit. One girl  reported longer bleeding duration than usual and one reported heavier bleeding. None experienced a reduction  in the amount nor the  length of menstrual  bleeding. AMH was not significantly altered following vaccination. |  |
| Morsi [61] | 2022 | Saudi Arabia | Cross-sectional | 100% Pfizer. 9.7% one dose, 90.3% two doses. | 731 female citizens and residents in Saudi Arabia | Range 16-40 years. 44% 20-29 years. | Online survey | • Duration of menstruation • Duration of the cycle • Amount of bleeding • Severity of pain | • 50.9% of the participants menstrual change, in particular, those who received the 2 doses. There was a significant and positive correlation between the number of doses and the experience of menstrual changes being associated with the 2 dosed women.  • 60.5% menstrual delay  • 30.4% early onset  • 9.1% menstrual change other than in the date.  • 43.3% decrease in the amount of menstrual flow  • 34.9 % increase in the amount of menstrual flow  • 62.4% increase in severity of pain  • 11% decrease in severity of pain.  • 60.8% of the respondents mentioned the persistence of the menstrual changes each following cycle. Changes were positively correlated with the age and negatively associated with the marital status being less in married. |  |
| Muhaidat [62] | 2022 | Jordan, UAE, KSA, Kuwait, Qatar, Turkey, Palestine, Iraq, Lebanon, Egypt, Oman, Morocco, Al-Bahrain, Tunisia, Sudan, and Syria. | Cross-sectional | 48.4% Pfizer-BioNTech, 35.3% Sinopharm, 13.4% AstraZeneca. 85% received two doses. | 2,269 females residing within the Middle East and North Africa | 34.3 years (SD 8.5), range 14-54 years. | Online survey | • Irregular menstruation • Menstrual cramps • Increased period frequency • Menorrhagia • Increase duration of the menstruation • Menstruation has stopped • Worsening of premenstrual symptoms • Intermenstrual bleeding | • About 66.3% of participants reported menstrual symptoms post-vaccination. Of those, symptoms appeared after a week in 30.5%, and within a month in 86.8%. 46.7% after first dose, 32.4% after second dose, 20.9% after both doses. In 93.6% of participants, the symptoms resolved within 2 months. • There was a significant relationship with country of residence (p < 0.001), irregular cycles (p < 0.001), smoking (p < 0.001), menstrual abnormalities during the COVID-19 pandemic (p < 0.001), negative impact on quality of life (p < 0.001), symptoms of COVID-19 vaccine general symptoms (p < 0.001), objective severity grade for vaccine general side effects (p < 0.001), and severity of COVID-19 infection (p = 0.006), and menstrual abnormalities. • There was a significantly higher prevalence of menstrual abnormalities among those who also experienced other adverse effects associated with the COVID-19 vaccination, including fever, fatigue, headache, nausea, and arm pain (17.6% vs 82.4%) (p < 0.001). In addition, there was a statistically significant association with the objective severity grade for vaccine general side effects (p < 0.001). • When comparing AstraZeneca, Sinopharm and Pfizer, differences in the incidence of menstrual abnormalities were statistically insignificant, 68.4%, 66.2%, 65.4%, respectively (p > 0.05).  • Mean duration of menstruation as reported by respondents had significantly increased from 6 ± 0.03 days pre-vaccine to 6.5 ± 0.1 post-vaccine (p < 0.001).  • Moreover, participants mean menstrual cycle length had significantly increased from 27 ± 6 days prior to taking the vaccine to 28.1 ± 10 days after being vaccinated (p < 0.001). |  |
| Namiki [63] | 2022 | Japan | Cross-sectional | 100% Pfizer/BioNTech | 309 Japanese premenopausal females with medical backgrounds from a single institution | Mean 31.9 years (SD 10.9) | Online survey | • Abnormal bleeding • Irregular menstrual cycle | • Abnormal bleeding 0.6%, 1.0%, and 3.0% for the ﬁrst, second, and third doses, respectively.  • Irregular menstrual cycle 1.9%, 4.9%, and 6.6% for the ﬁrst, second, and third doses, respectively.  • The effects of COVID-19 vaccination on menstruation seem limited. Abnormal bleeding occurred more commonly after the third dose than after the ﬁrst dose (p = 0.047). An irregular menstrual cycle was signiﬁcantly more common after the second and third doses than after the ﬁrst dose (p = 0.06 for each). | • This is the ﬁrst report to investigate adverse events after the third vaccination. |
| Qashqari [64] | 2022 | Saudi Arabia | Cross-sectional | 72.5% Pfizer, 26.8% AstraZeneca, 0.7% Moderna (first dose). 82,6% Pfizer, 15.6% AstraZeneca, 1.9% Moderna (second dose, n=1606, 68.7%) | 2338 females in Saudi Arabia | Mean 35.4 years (SD 9.5). Aged 12 and above. | Online survey | • Abnormal bleeding • Irregular menstrual cycle • Menstrual cramps • Premenstrual syndrome | • After first dose: period was heavier than usual (14.5%), later than usual (27.7%), cramps were worse than usual (26.5%), and premenstrual syndrome was worse than usual (23.7%).  • After second dose: period was heavier than expected (17.1%), later than usual (24.7%), and cramps were worse than usual (26.8%).  • The premenstrual syndrome was worse than usual among 24.0% of women, indicating that menstrual problems following vaccination significantly impacted the quality of life of the participating females.  • No significant associations were found between the type of COVID-19 vaccine and the impact on the menstrual cycle, either for the first or second dose (Pvalues > 0.05).  • A significant association was found only between the first dose vaccination day and the impact on the menstrual cycle in the second question of “After receiving the COVID-19 vaccine, your next period was” (P-value ≤ 0.05).  • Significant associations were found between the  second dose vaccination day and the impact on the menstrual cycle in the first and second questions of “After receiving the COVID-19 vaccine, your next period was”, and “After receiving the first dose, your next period was," respectively (P-values ≤ 0.05). |  |
| Qazi [65] | 2023 | India | Cross-sectional | 70% AstraZeneca (Covishield), 30% Covaxin (Bharat Biotech) | 300 women of reproductive age (15–49 years). | Mean 26.2 ± 4.8 years (96% 20-39 years) | Online survey | • Change in regularity of menstrual cycle  • Change in cycle duration  • Menstrual cramps or pain  • Change in menstrual flow | • 30 participants (10%) reported a change in the regularity of menstruation and 33 (11%) participants reported a change in cycle duration after vaccination. Six (33%) reported cramps along with pain after vaccination. 74 (25%) reported a change in menstrual flow after vaccination.  • A statistically significant association was found between the type of vaccination received and regularity of cycles (P < 0.001), a change in the duration of the cycle (P < 0.001), a change in intensity of pain after vaccination (P = 0.005),  and a change in menstrual flow after vaccination (P < 0.001). There was no significant association between the type  of COVID-19 vaccination and the change in the duration of the pain after vaccination (P =  0.149). |  |
| Rastegar [66] | 2023 | Iran | Cross-sectional | Sinopharm (64% first dose, 64% second dose, 46% third dose), AstraZeneca (21% first dose, 21% second dose, 30% third dose), Sputnik (6% first dose, 6% second dose, 1% third dose), Barkat (4% first dose, 4% second dose, 3% third dose), Bharat (2% first dose, 1% second dose, 0.3% third dose) | 455 women aged 15-55 years | 38% 31-40 years | Online survey | • Premature menstruation  • Latency of menstruation  • Heavy bleeding  • Spot bleeding  • Increase/decrease the number of menstrual days  • Dysmenorrhea | • 18.4% of persons have menstrual disturbance after receiving ﬁrst dose of vaccine, and 20.9% after second dose, 12.5% following third dose and 0.2% after all three doses.  Latency of menstruation: 19.4%  Premature menstruation: 17.1% |  |
| Rodriquez Quejada [67] | 2022 | Colombia | Cross-sectional | 43.57% Pfizer,. 18% Sinovac, 15.68% J&J/Janssen, 12.31% Moderna, 6% AstraZeneca, 4.42% others. | 408 women | Range 18-41 years, median 28 years. | Online survey | • Menstrual frequency • Menstrual regularity • Menstrual duration • Menstrual volume | • 184 women (45%) reported alterations in the menstrual cycle. Frequency (normal 43.47%, infrequent 25%, and frequent 31.53%), regularity (regular 51.08%, irregular 42.93%, and absent/amenorrhea 5.97%), duration (normal 65.21%, prolonged 26.08%, absent/amenorrhea 8.69%), and volume (heavy 41.84%, light 20.65%, and absent/amenorrhea 6.52%). |  |
| Rogers [68] | 2022 | United Kingdom | Prospective cohort study (VAC4COVID) | 47.9% ChadOx1 (two doses), 36.9% BNT162b2 (two doses), 9.9% other (any doses) | 11,475 UK residents, 59.7% female | Mean 54.8 years | Online survey | • Changes in menstrual symptoms (including menstrual cycle alteration or intermenstrual bleeding, heavy bleeding, or painful periods/cramping) | • 0.3% menstrual symptoms after vaccination.  • No differences between vaccine type or dose order were detected.  • Unadjusted percentages of reporting menstrual symptoms, including menstrual cycle alteration or intermenstrual bleeding (12 events), heavy bleeding (11) or painful periods/cramping (5) within 12 weeks of vaccination were higher after BNT162b2 vaccinations (0.6% after first dose, 0.4% after second dose) than after ChAdOx1 (0.2% after first dose, 0.2% after second dose). However, there was no difference between vaccines after adjusting for age in a proportional hazards model and overall cumulative rates were low. Participants reported these events as 25% mild, 54% moderate and 21% severe; none resulted in hospitalisation. |  |
| Saleh Alzahrani [69] | 2023 | Saudi Arabia | Cross-sectional | Pfizer-BioNTech (79.8% first dose, 79.0% second dose, 47.2% third dose), Oxford-AstraZeneca (18.9% first dose, 16.1% second dose, 0.5% third dose), Moderna (0.8% first dose, 3.8% second dose, 12.4% third dose). | 1,066 females aged 18-50 years who lived in Riyadh, Saudi Arabia | 64% 19-29 years | Online survey | • Irregular menstruation  • Increase/decrease time between one cycle and the next cycle  • Increase/decrease amount of blood  • Decrease/increase number of days of bleeding  • Absence of the cycle after taking the vaccine | • Irregular menstruation: 12.0%  • Increased time between one cycle and the next cycle: 7.5%  • Abnormal menstruation was signiﬁcantly associated with hypertension (p-value = 0.024), polycystic ovary syndrome (p-value = 0.001), and at least one gynaecological condition (p-value < 0.001). However, the multivariate regression analysis showed that menstrual abnormality before receiving the COVID-19 vaccine (OR = 0.09, 95% CI, 0.06 to 0.14, p-value < 0.001) was more likely to be associated with abnormal menstruation after receiving the vaccine.  • The types of the vaccine were not associated with menstrual abnormality. |  |
| Sarfraz [70] | 2022 | Worldwide (38.2% India, 34.9% Pakistan, 13.1% U.S.A.) | Cross-sectional | 65% two doses. Vaccine types not reported. | 510 female respondents (61.6% healthcare workers), 493 (96.7%) vaccinated | N.R. | Online survey | • Changes in the length of cycle between periods • Periods sooner or later  • Heavier or lighter bleeding during periods • Bleeding for more or fewer days | • Changes in the length of cycle between periods (yes: 18.2%, no: 81.1%, p<0.001) • Periods sooner or later (later: 18%, sooner: 10.2%, p=0.024) • Heavier or lighter bleeding during periods (heavier: 9.8%, lighter: 14.5%, p<0.001) • Bleeding for more or fewer days (fewer days: 11.4%, more days: 7.1%, p<0.001) • Vaccinated compared to non-vaccinated women had a higher risk of change in inter-cycle length between periods (OR = 3.172; 95% CI = 0.470–21.431). |  |
| Sualeh [71] | 2022 | Pakistan | Cross-sectional | N.R. | 384 females aged 18 years and above, of which 146 (38%) vaccinated | Median 21 (IQR 2) years | Online survey | • Cycle duration  • Menstrual flow • Menstrual symptoms | • The difference between the post-vaccine menstruation affected (n=146) and the unaffected cohort (n=238) was significant. • For every increase in PSS score, vaccinated participants were 1.04 times more likely to have an impact on their menstruation (OR=1.04, 95% CI 1.01-1.08). Furthermore, participants with strenuous physical activity were 5.63 times more likely to experience post-vaccination menstrual changes (OR=5.63, 95% CI 1.32-23.98). In contrast, participants with normal pre-vaccination menstrual flow were 0.12 times less likely to experience any post-vaccine menstrual change (OR=0.12, 95% CI 0.02-0.56). • Cycle duration (21% increased, 21% decreased) • Menstrual flow (13% scantier, 15% heavier) • Menstrual symptoms (9% better, 35% worse) Women having normal prevaccination menstrual flow were less likely to experience post-vaccine menstrual changes. The significant results of this study pointed more toward the association of physical activity and stress with menstrual cycle, with COVID-19 vaccine being an effect modifier. |  |
| Taşkaldıran [72] | 2022 | Turkey | Descriptive, cross-sectional | 82.3% Pfizer-BioNTech, 7.1% Sinovac (CoronaVac), 9.4% both Pfizer-BioNTech and Sinovac (CoronaVac), and 1 person AstraZeneca | 537 vaccinated women who were admitted to the hospital or worked in the hospital. | Mean 23.34 years. Range 18-50 years. | Face-to-face survey | • Length of the menstrual cycle • Duration of the period • Amount of bleeding • Intermenstrual bleeding | • 82 (15.1%) changes in their menstrual patterns after vaccination. 68 of those received the Pﬁzer-BioNTech vaccine, 12 received the Pﬁzer-BioNTech and Sinovac (CoronaVac) vaccine, and 2 received the Sinovac (CoronaVac) vaccine.  • Of the participants with a change in the menstrual pattern, 43.3% after 2nd dose, 33.3% after 1st dose, 21.7% after 3rd dose, and 1.7% after 4th dose.  • Of the participants with altered menstrual patterns, 74.2% stated that these menstrual pattern changes did not recur in the following vaccinations. • The changes in the menstrual pattern were as follows: shorter cycle in 20 (3.7%) patients (early menstruation), delayed cycle in 31 (5.7%) patients (late menstruation), intermenstrual bleeding in 13 (2.4%) patients, heavier menstrual bleeding in 20 (3.7%) patients, lighter menstrual bleeding in 14 (2.6%) patients, shorter period in 13 (2.4%) patients, and longer period in 16 (2.9%) patients. |  |
| Trogstad [14] | 2022 | Norway | Self-controlled case series, as part of the Norwegian Young Adult Cohort | Comirnaty 1st dose 57.9%, Spikewax 1st dose 35.5%, other 5%. Comirnaty 2nd dose 43.5%, Spikewax 48.1%, other 0%. | 5,688 women (98.4% vaccinated) | Range 18-30 years | Mobile-phone questionnaires | • More heavy bleeding than usual • Longer lasting menstruation • Shorter interval between menstruations • Longer interval between menstruations • Spot bleedings between menstruations • Stronger pain during menstruation • Period pain without bleeding • Any other symptom from the pelvic region | • Menstrual disturbances were generally common, with a prevalence close to 40 per cent, regardless of vaccination. • The relative risk of more heavy bleeding than usual during the exposed compared to unexposed period for first dose vaccination was 1.90 (95% CI: 1.69-2.13), while it was 1.84 (1.66-2.03) for the second dose.  • Significant increase in menstrual disturbances after vaccination, particularly for heavier bleeding than usual, longer duration and for short interval between menstruations. • On average, menstrual disturbances after the first dose returned to normal by the time the second vaccination was given, approximately two months after the first dose, suggesting that in most cases the menstrual disturbances associated with the first vaccination were transient. | • Participants were randomly drawn from the National Population Registry, this minimizes the chance of selection bias. |
| Velasco-Regulez [73] | 2022 | Spain | Self-controlled case series | N.R. | 371 app users with at least 5 consecutive cycles | 18 to 24, 11.85%; 25 to 34, 49.15%; 35 to 44, 28.56%; 45 to 54, 8.31%; other, 2.13%. | Menstrual cycle tracking smartphone application Lunar App | • Menstrual cycle length • Menses length change • Variations in the usual blood quantity • Pain intensity during menses | • Increase in the median cycle length of 0.5 (0.0-1.0) days (P value <0.005) for all individuals, with 8.08% of the individuals having an increase of 8 or more days, which is considered clinically signiﬁcant. • No variation in menses length. • No signiﬁcant variations in the percentages of cycles with abnormal blood loss or pain intensity. • The stratiﬁed analysis showed an association between the phase of the menstrual cycle of the individual at vaccination time and cycle length change.  • Individuals vaccinated during the follicular phase showed a median cycle length increase of 1 (0.0-1.0) day (P value <.005), with 11.82% of the users having an increase of 8 or more days. Individuals vaccinated during the luteal phase showed no change. |  |
| Wali [74] | 2023 | Saudi Arabia | Cross-sectional | First dose: 74% Pfizer, 25% Oxford-AstraZeneca, 1% Moderna.  Second dose: 76% Pfizer, 19% Oxford-AstraZeneca, %% Moderna.  Third dose: 81% Pfizer | 297 women between 15 and 50 years | 48% 21-32 years | Online survey | • Irregular menstrual period  • Days of bleeding on average during the period  • Heavy bleeding or not  • Painful period or not  • Missed period  • Change in premenstrual symptoms | • 44% reported a change in the length and amount of the menstrual cycle, and 29% worsened premenstrual syndrome (PMS).  There was no significant association between the type and the number of doses on menstrual alterations. |  |
| Wang [75] | 2022 | United States of America and Canada | Prospective cohort study, part of the Nurses' Health Study | 89% Pfizer or Moderna, 2% Janssen, 9% unvaccinated | 3,527 vaccinated premenopausal nurses in the Nurses' Health Study vs. 331 unvaccinated | Mean 33.4 (SD 6.3) years | Questionnaires | • Change in cycle length (shorter/longer) • Change in cycle regularity (more regular/less regular) | • Vaccinated women had a higher risk of increased cycle length than unvaccinated women (odds ratio, 1.48; 95% conﬁdence interval, 1.00-2.19), after adjusting for sociodemographic and behavioral factors.  • These associations were similar after in addition accounting for pandemic-related stress.  • COVID-19 vaccination was only associated with change to longer cycles in the ﬁrst 6 months after vaccination (0-6 months: odds ratio, 1.67 [95% conﬁdence interval, 1.05-2.64]; 7-9 months: odds ratio, 1.43 [95% conﬁdence interval, 0.96-2.14]; >9 months: odds ratio, 1.41 [95% conﬁdence interval, 0.91-2.18]) and among women whose cycles were short, long, or irregular before vaccination (odds ratio, 2.82 [95% conﬁdence interval, 1.51-5.27]; odds ratio, 1.10 [95% conﬁdence interval, 0.68-1.77] for women with normal length, regular cycles before vaccination). Messenger RNA and adenovirus-vectored vaccines were both associated with this change.  • COVID-19 vaccination may be associated with a short-term change toward longer menstrual cycles. | • Periodic surveys were administered over a 1year period during the COVID-19 pandemic, rigorously measuring incident SARS-CoV-2 infection and COVID-19 vaccination, allowing comparison with those uninfected and unvaccinated. • Able to control for the impacts of the pandemic on an in- dividual’s social functioning, mental health, and behavioral practices using validated measures.  • Menstrual cycle characteristics were collected prospectively throughout women’s reproductive years before and during the COVID-19 pandemic, which allowed the researchers to compare menstrual cycle characteristics before and after COVID-19 infection and vaccination. |
| Wesselink [76] | 2023 | United States of America and Canada | Cohort study | 32.3% Moderna Spikevax, 60.9% Pfizer-BioNTech, 6.6% Janssen (Johnson & Johnson), 0.2% AstraZeneca | 1,137 female participants who enrolled in Pregnancy Study Online (PRESTO), aged 21-45, resided in the U.S. or Canada, and were trying to conceive without the use of fertility  treatment.  437 received at least one COVID-19 vaccine | Mean 31.4 years | Online survey | • Cycle regularity  • Cycle length  • Bleed length  • Heaviness  of bleed  • Menstrual pain | • Participants had 1.1 day longer menstrual cycles after receiving the ﬁrst dose of COVID-19 vaccine (95 % CI: 0.4, 1.9) and 1.3 day longer cycles after receiving the second dose (95 % CI: 0.2, 2.5).  • No strong associations between COVID-19 vaccination and cycle regularity, bleed length, heaviness of bleed, or menstrual pain.  • Small and temporary increase in cycle length following COVID-19 vaccination, but little difference in cycle regularity, bleed length, heaviness of bleed, or menstrual pain. The ﬁrst menstrual cycle after each COVID-19 vaccine dose was approximately 1 day longer on average than menstrual cycles before vaccination, but returned to the prevaccination length at the second cycle. The prevalence of long cycles (>38 days) was also slightly higher immediately after vaccination, but returned to baseline by the following cycle. |  |
| Wong [77] | 2022 | United States of America | Observational cohort study | 49.8% Pfizer BNT162b2, 46.8% Moderna mRNA-1273, 4.8% Johnson&Johnson Ad26.COV2.S, <0.1% other/unknown. 82.6% 1 dose, 70.6% 2 doses, 12.5% >=3 doses | 5,975,363 women in an active vaccine safety monitoring system, v-safe. 62,679 women (1%) reported menstrual irregularities or vaginal bleeding. | Range 18-85+ years | Online survey | • Menstrual irregularities or vaginal bleeding • Timing of the menses • Severity of menstrual symptoms • Menopausal bleeding • Resumption of menses | • Common themes identified included timing of menstruation (70,981 [83·6%] responses, 52% Pfizer, 42% Moderna) and severity of menstrual symptoms (56 890 [67·0%] responses, 48.9% Pfizer, 44.7% Moderna). Other themes included menopausal bleeding (3439 [4·0%] responses, 48.9% Pfizer, 44.7% Moderna) and resumption of menses (2378 [2·8%] responses, 50.7% Pfizer, 42.5% Moderna). • Among the 63,815 respondents who reported menstrual irregularities or vaginal bleeding, most respondents received BNT162b2 (33 149 [51·9%] respondents) or mRNA-1273 (26,741 [41·9%] respondents) vaccines. | • Menstrual irregularities and vaginal bleeding after COVID-19 vaccination are being reported, although this study is unable to assess whether these events are caused by COVID-19 vaccination. |
| Woon [78] | 2022 | United Kingdom | Prospective cohort study | 82% Pfizer, 14% Moderna, 4% AstraZeneca | 79 individuals who regularly experience either menstrual periods or withdrawal bleeding as a result of breaks in taking hormonal contraception. | Median 30 years (IQR 27-35) | Daily journals | • Menstrual timing • Menstrual flow | • Either dose of the COVID19 vaccine is associated with a delay to the subsequent period in spontaneously cycling participants (2.3 days after dose 1 (p=0.0045); 1.3 days after dose 2, p=0.041) but this change rapidly reverses.  • No change to timing was detected in those on hormonal contraception.  • No change in menstrual flow associated with either dose of the vaccine, in either spontaneously cycling participants or those on hormonal contraception.  • No association between menstrual changes and other commonly-reported side effects of vaccination, such as sore arm, fever and fatigue. |  |
| Zhang [79] | 2022 | China | Cross-sectional | 73% Pfizer-Biontech, 21% Moderna, 6% Janssen | 14,431 reports of menstrual disorder, of which 13,118 were associated with COVID-19 vaccine | Median 36 years (30.0-43.0) | Vaccine Adverse Event Reporting System (VAERS) | • Menstruation irregular  • Menstruation delayed  • Menstrual disorder  • Metrorrhagia  • Amenorrhoea • Menorrhagia  • Hypomenorrhoea  • Intermenstrual bleeding | • The ROR was 7.83 (95% confidence interval [95%CI]: 7.39–8.28). The most commonly reported event in the COVID-19 vaccinated group was Menstruation irregular (4626 reports, 35%), and a higher percentage of female aged 30–49 years reported menstrual disorders (42.55%) after exposure to COVID-19 Vaccines. Menstruation delayed (21%), menstrual disorder (15%), Metrorrhagia (13%), amenorrhoea (12%), menorrhagia (0.2%), hypomenorrhoea (8%), intermenstrual bleeding (16%). Both for all reports of menstrual disorders (ROR = 5.82; 95%CI: 4.93–6.95) and excluding reports of unknown age (ROR = 13.02; 95%CI: 10.89–15.56),suggest that female age may be associated with menstrual disorders after vaccination with the COVID-19 Vaccines. |  |

# REFERENCES SUPPLEMENTAL FILES

**[**1] Danish Medicines Agency. No evidence that menstrual disorders are triggered by COVID-19 vaccination 2021 [Accessed 13 June 2023]. Available from: <https://laegemiddelstyrelsen.dk/en/news/2021/no-evidence-that-menstrual-disorders-are-triggered-by-covid-19-vaccination/>.

[2] European Medicines Agency. Signal assessment report on heavy menstrual bleeding with tozinameran / Comirnaty (COVID-19 mRNA vaccine) 2022 [Accessed 14 June 2023]. Available from: <https://www.ema.europa.eu/en/documents/prac-recommendation/signal-assessment-report-heavy-menstrual-bleeding-tozinameran/comirnaty-covid-19-mrna-vaccine_en.pdf>.

[3] European Medicines Agency. Signal assessment on heavy menstrual bleeding with COVID-19 mRNA vaccine (Spikevax) 2022 [Accessed 14 June 2023]. Available from: <https://www.ema.europa.eu/en/documents/prac-recommendation/signal-assessment-heavy-menstrual-bleeding-covid-19-mrna-vaccine-spikevax_en.pdf>.

[4] Norwegian Medicines Agency. Reported suspected adverse reactions to COVID-10 vaccines as of 01.03.2022 2022 [Accessed 21 June 2023]. Available from: <https://legemiddelverket.no/Documents/English/Covid-19/20220307%20Reported%20suspected%20adverse%20reactions%20coronavirus%20vaccines.pdf>.

[5] legemiddelverk S. Reports of suspected adverse reactions to coronavirus vaccine as of 22 June 2021 2021 [Accessed 21 June 2023]. Available from: <https://legemiddelverket.no/nyheter/reports-of-suspected-adverse-reactions-to-coronavirus-vaccine-as-of-22-june-2021>.

[6] Medicines and Healthcare products Regulatory Agency. COVID-19 vaccines: updates for August 2021 [Accessed 21 June 2023]. [Available from: <https://www.gov.uk/drug-safety-update/covid-19-vaccines-updates-for-august-2021>.

[7] Male V. Menstrual changes after covid-19 vaccination. Bmj. 2021;374:n2211.

[8. Merchant H. CoViD-19 post-vaccine menorrhagia, metrorrhagia or postmenopausal bleeding and potential risk of vaccine-induced thrombocytopenia in women. BMJ. 2021;373:n958.

[9] National Institute of Child Health and Human Development. NIH funds studies to assess potential effects of COVID-19 vaccination on menstruation 2021 [Accessed 14 June 2023]. Available from: <https://www.nichd.nih.gov/newsroom/news/083021-COVID-19-vaccination-menstruation>.

[10] Alghamdi AN, Alotaibi MI, Alqahtani AS, Al Aboud D, Abdel-Moneim AS. BNT162b2 and ChAdOx1 SARS-CoV-2 Post-vaccination Side-Effects Among Saudi Vaccinees. Front Med (Lausanne). 2021;8:760047.

[11] Netherlands Pharmacovigilance Centre Lareb. Menstrual disorders and postmenopausal bleeding after administration of COVID-19 vaccines 2021 [Accessed 14 June 2023]. Available from: <https://www.lareb.nl/media/uoneih5z/signals_2021_menstrual_disorders-and-postmenopausal_bleeding-and-covid-19-vaccines.pdf>.

[12] Netherlands Pharmacovigilance Centre Lareb. Overview menstrual disorders after Covid-19 vaccination – Update 2022 [Accessed 14 June 2023]. Available from: <https://www.lareb.nl/media/dxcjbjmv/signals_2022_covid19-vaccines-and-menstrual-disorders_update.pdf>.

[13] Edelman A, Boniface ER, Benhar E, Han L, Matteson KA, Favaro C, et al. Association Between Menstrual Cycle Length and Coronavirus Disease 2019 (COVID-19) Vaccination: A U.S. Cohort. Obstet Gynecol. 2022;139(4):481-9.

[14] Trogstad L, Laake I, Robertson AH, Mjaaland S, Caspersen IH, Juvet LK, et al. Increased occurrence of menstrual disturbances in 18- to 30-year-old women after COVID-19 vaccination [pre-print]. 2022.

[15] European Medicines Agency. Meeting highlights from the Pharmacovigilance Risk Assessment Committee (PRAC) 7 - 10 February 2022 2022 [Accessed 14 June 2023]. Available from: <https://www.ema.europa.eu/en/news/meeting-highlights-pharmacovigilance-risk-assessment-committee-prac-7-10-february-2022>.

[16] European Medicines Agency. Meeting highlights from the Pharmacovigilance Risk Assessment Committee (PRAC) 7-10 June 2022 2022 [Accessed 14 June 2023. Available from: <https://www.ema.europa.eu/en/news/meeting-highlights-pharmacovigilance-risk-assessment-committee-prac-7-10-june-2022>.

[17] Duijster JW, Schoep ME, Nieboer TE, Jajou R, Kant A, Van Hunsel F. Menstrual abnormalities after COVID-19 vaccination in the Netherlands: a description of spontaneous and longitudinal patient-reported data. Br J Clin Pharmacol. 2023.

[18] European Medicines Agency. Meeting highlights from the Pharmacovigilance Risk Assessment Committee (PRAC) 24 - 27 October 2022 2022 [Accessed 14 June 2023]. Available from: <https://www.ema.europa.eu/en/news/meeting-highlights-pharmacovigilance-risk-assessment-committee-prac-24-27-october-2022>.

[19] European Medicines Agency. COVID-19 vaccines safety update 2022 [Accessed 14 June 2023]. Available from: <https://www.ema.europa.eu/en/documents/covid-19-vaccine-safety-update/covid-19-vaccines-safety-update-10-november-2022_en.pdf>.

[20] Medicines and Healthcare products Regulatory Agency. Coronavirus vaccine - summary of Yellow Card reporting 2022 [Accessed 14 June 2023]. Available from: <https://www.gov.uk/government/publications/coronavirus-covid-19-vaccine-adverse-reactions/coronavirus-vaccine-summary-of-yellow-card-reporting>.

[21] European Medicines Agency. COMIRNATY - Procedural steps taken and scientific information after the authorisation 2022 [Accessed 14 June 2023]. Available from: <https://www.ema.europa.eu/en/documents/procedural-steps-after/comirnaty-epar-procedural-steps-taken-scientific-information-after-authorisation_en.pdf>

[22] European Medicines Agency. Spikevax - Procedural steps taken and scientific information after the authorisation 2022 [Accessed 14 June 2023]. Available from: <https://www.ema.europa.eu/en/documents/procedural-steps-after/spikevax-previously-covid-19-vaccine-moderna-epar-procedural-steps-taken-scientific-information_en.pdf>.

[23] Abdollahi A, Naseh I, Kalroozi F, Kazemi-Galougahi MH, Nezamzadeh M, Sabeti Billandi S, et al. Comparison of Side Effects of COVID-19 Vaccines: Sinopharm, AstraZeneca, Sputnik V, and Covaxin in Women in Terms of Menstruation Disturbances, Hirsutism, and Metrorrhagia: A Descriptive-Analytical Cross-Sectional Study. Int J Fertil Steril. 2022;16(3):237-43.

[24] Akarsu GD. Determining the Health Problems Experienced by Young Adults in Turkey, Who Received the COVID-19 Vaccine. Vaccines (Basel). 2022;10(9).

[25] Al-Furaydi A, Alrobaish SA, Al-Sowayan N. The COVID-19 vaccines and menstrual disorders. Eur Rev Med Pharmacol Sci. 2023;27(3):1185-91.

[26] L MMA-M, I AM, Khamaiseh K, S NA-B, Al-Kuran OAH. Short Term Effect of Corona Virus Diseases Vaccine on the Menstrual Cycles. Int J Womens Health. 2022;14:1385-94.

[27] Alahmadi AM, Aljohani AH, Fadhloun RA, Almohammadi AS, Alharbi DF, Alrefai LS. The Effect of the COVID-19 Vaccine on the Menstrual Cycle Among Reproductive-Aged Females in Saudi Arabia. Cureus. 2022;14(12):e32473.

[28] Aldali JA, Alotaibi FT, Alasiri GA, Almesned RA, Alromih AM, Almohandes AM, et al. Evaluate the side effect associated with COVID-19 vaccine on adolescents in Riyadh, Saudi Arabia: A cross-section study. Saudi Med J. 2022;43(11):1248-53.

[29] Alvergne A, Woon EV, Male V. Effect of COVID-19 vaccination on the timing and flow of menstrual periods in two cohorts. Front Reprod Health. 2022;4:952976.

[30] Alvergne A, Kountourides G, Argentieri MA, Agyen L, Rogers N, Knight D, et al. A retrospective case-control study on menstrual cycle changes following COVID-19 vaccination and disease. iScience. 2023;26(4):106401.

[31] Alvergne A, Boniface E, Darney B, Shea A, Weber K, Ventola C, et al. Associations Among Menstrual Cycle Length, Coronavirus Disease 2019 (COVID-19), and Vaccination. Obstet Gynecol. 2023.

[32] Amer AA, Amer SA, Alrufaidi KM, Abd-Elatif EE, Alafandi BZ, Yousif DA, et al. Menstrual changes after COVID-19 vaccination and/or SARS-CoV-2 infection and their demographic, mood, and lifestyle determinants in Arab women of childbearing age, 2021. Front Reprod Health. 2022;4:927211.

[33] Anjorin AA, Odetokun IA, Nyandwi JB, Elnadi H, Awiagah KS, Eyedo J, et al. Public Health Surveillance for Adverse Events Following COVID-19 Vaccination in Africa. Vaccines (Basel). 2022;10(4).

[34] Baena-García L, Aparicio VA, Molina-López A, Aranda P, Cámara-Roca L, Ocón-Hernández O. Premenstrual and menstrual changes reported after COVID-19 vaccination: The EVA project. Womens Health (Lond). 2022;18:17455057221112237.

[35] Barabás K, Makkai B, Farkas N, Horváth HR, Nagy Z, Váradi K, et al. Influence of COVID-19 pandemic and vaccination on the menstrual cycle: A retrospective study in Hungary. Front Endocrinol (Lausanne). 2022;13:974788.

[36] Bisgaard Jensen C, Bech BH, Hansen SN, Rask CU, Fink P, Nielsen H, et al. Prevalence of and risk factors for self-reported menstrual changes following COVID-19 vaccination: a Danish cohort study. Hum Reprod. 2023.

[37] Caspersen IH, Juvet LK, Feiring B, Laake I, Robertson AH, Mjaaland S, et al. Menstrual disturbances in 12- to 15-year-old girls after one dose of COVID-19 Comirnaty vaccine: Population-based cohort study in Norway. Vaccine. 2023;41(2):614-20.

[38] Cheng Y, Li T, Zheng Y, Xu B, Hu Y, Zhou YH. Self-Reported adverse events among Chinese healthcare workers immunized with COVID-19 vaccines composed of inactivated SARS-CoV-2. Human Vaccines & Immunotherapeutics. 2022;18(5):e2064134.

[39] Chiang MR, Shih LC, Lu CC, Fang SH. The COVID-19 vaccine did not affect the basal immune response and menstruation in female athletes. Physiol Rep. 2023;11(3):e15556.

[40] Dabbousi AA, El Masri J, El Ayoubi LM, Ismail O, Zreika B, Salameh P. Menstrual abnormalities post-COVID vaccination: a cross-sectional study on adult Lebanese women. Ir J Med Sci. 2022:1-8.

[41] Dar-Odeh N, Abu-Hammad O, Qasem F, Jambi S, Alhodhodi A, Othman A, et al. Long-term adverse events of three COVID-19 vaccines as reported by vaccinated physicians and dentists, a study from Jordan and Saudi Arabia. Hum Vaccin Immunother. 2022;18(1):2039017.

[42] Darney BG, Boniface ER, Van Lamsweerde A, Han L, Matteson KA, Cameron S, et al. Impact of coronavirus disease 2019 (COVID-19) vaccination on menstrual bleeding quantity: An observational cohort study. Bjog. 2023;130(7):803-12.

[43] Dellino M, Lamanna B, Vinciguerra M, Tafuri S, Stefanizzi P, Malvasi A, et al. SARS-CoV-2 Vaccines and Adverse Effects in Gynecology and Obstetrics: The First Italian Retrospective Study. Int J Environ Res Public Health. 2022;19(20).

[44] Edelman A, Boniface ER, Male V, Cameron ST, Benhar E, Han L, et al. Association between menstrual cycle length and covid-19 vaccination: global, retrospective cohort study of prospectively collected data. BMJ Med. 2022;1(1).

[45] El-Shitany NA, Bagher AM, Binmahfouz LS, Eid BG, Almukadi H, Badr-Eldin SM, et al. The Adverse Reactions of Pfizer BioNTech COVID-19 Vaccine Booster Dose are Mild and Similar to the Second Dose Responses: A Retrospective Cross-Sectional Study. Int J Gen Med. 2022;15:6821-36.

[46] Farah S, Hijazi M, Aoun E, Boueri M, Nasr E, Chlala W, et al. Effect of COVID-19 vaccinations on menstrual cycle and postmenopausal bleeding among health care workers: A cross-sectional study. Int J Gynaecol Obstet. 2023;162(2):532-40.

[47] Farhat M, Al-Ibrahim R, Almohammedali A, Aljishi R, Alalwan B. Study of the Side Effects of Pfizer and Oxford COVID-19 Vaccines in the Eastern Province of Saudi Arabia. Int J Gen Med. 2022;15:7547-58.

[48] Farland LV, Khan SM, Shilen A, Heslin KM, Ishimwe P, Allen AM, et al. COVID-19 vaccination and changes in the menstrual cycle among vaccinated persons. Fertil Steril. 2022;119(3):392-400.

[49] Filfilan NN, Bukhari S, Rizwan M, Bukhari NM, Aref NK, Arain FR, et al. Effects of Different Types of COVID-19 Vaccines on Menstrual Cycles of Females of Reproductive Age Group (15-49): A Multinational Cross-Sectional Study. Cureus. 2023;15(5):e39640.

[50] Gibson EA, Li H, Fruh V, Gabra M, Asokan G, Jukic AMZ, et al. Covid-19 vaccination and menstrual cycle length in the Apple Women's Health Study. medRxiv. 2022.

[51] Hariton E, Morris JR, Ho K, Chen C, Cui E, Cedars MI. The effect of the coronavirus disease 2019 vaccine and infection on menstrual cycle length: an analysis of 12 months of continuous menstrual cycle data from 5,314 participants. Fertil Steril. 2023.

[52] Issakov G, Tzur Y, Friedman T, Tzur T. Abnormal Uterine Bleeding Among COVID-19 Vaccinated and Recovered Women: a National Survey. Reprod Sci. 2022:1-9.

[53] Kajiwara S, Akiyama N, Baba H, Ohta M. Association between COVID-19 vaccines and the menstrual cycle in young Japanese women. J Infect Chemother. 2023.

[54] Laganà AS, Veronesi G, Ghezzi F, Ferrario MM, Cromi A, Bizzarri M, et al. Evaluation of menstrual irregularities after COVID-19 vaccination: Results of the MECOVAC survey. Open Med (Wars). 2022;17(1):475-84.

[55] Lee KMN, Junkins EJ, Luo C, Fatima UA, Cox ML, Clancy KBH. Investigating trends in those who experience menstrual bleeding changes after SARS-CoV-2 vaccination. Sci Adv. 2022;8(28):eabm7201.

[56] Lessans N, Rottenstreich A, Stern S, Gilan A, Saar TD, Porat S, et al. The effect of BNT162b2 SARS-CoV-2 mRNA vaccine on menstrual cycle symptoms in healthy women. Int J Gynaecol Obstet. 2023;160(1):313-8.

[57] Ljung R, Xu Y, Sundström A, Leach S, Hallberg E, Bygdell M, et al. Association between SARS-CoV-2 vaccination and healthcare contacts for menstrual disturbance and bleeding in women before and after menopause: nationwide, register based cohort study. Bmj. 2023;381:e074778.

[58] Matar SG, Nourelden AZ, Assar A, Bahbah EI, Alfryjat AM, Hasabo EA, et al. Effect of COVID-19 vaccine on menstrual experience among females in six Arab countries: A cross sectional study. Influenza Other Respir Viruses. 2023;17(1):e13088.

[59] Mínguez-Esteban I, García-Ginés P, Romero-Morales C, Abuín-Porras V, Navia JA, Alonso-Pérez JL, et al. Association between RNAm-Based COVID-19 Vaccines and Permanency of Menstrual Cycle Alterations in Spanish Women: A Cross-Sectional Study. Biology (Basel). 2022;11(11).

[60] Mohr-Sasson A, Haas J, Sivan M, Zehori Y, Hemi R, Orvieto R, et al. The effects of Covid-19 mRNA vaccine on adolescence gynecological well-being. Arch Gynecol Obstet. 2023;307(5):1625-31.

[61] Morsi AA, Mersal EA, Hassanein AM, Alshammri A, Alshammari A, Alkahmous N, et al. The Association Between COVID-19 Pfizer Vaccine and The Reported Post-Vaccination Menstrual Changesi Citizen and Resident Women in KSA: Results of Riyadh Survey Study The Egyptian Journal of Hospital Medicine. 2022;87:1442-8.

[62] Muhaidat N, Alshrouf MA, Azzam MI, Karam AM, Al-Nazer MW, Al-Ani A. Menstrual Symptoms After COVID-19 Vaccine: A Cross-Sectional Investigation in the MENA Region. Int J Womens Health. 2022;14:395-404.

[63] Namiki T, Komine-Aizawa S, Takada K, Takano C, Trinh QD, Hayakawa S. The association of three doses of the BNT162b2 mRNA vaccine with abnormal bleeding and an irregular menstrual cycle among premenopausal females: A single institute observation study. J Obstet Gynaecol Res. 2022;48(11):2903-10.

[64] Qashqari FSI, Dahlawi M, Assaggaf HM, Alsafi R, Gari A, Abudawood A, et al. Effect of the COVID-19 Vaccine on the Menstrual Cycle among Females in Saudi Arabia. Ethiop J Health Sci. 2022;32(6):1083-92.

[65] Qazi TB, Dkhar SA, Quansar R, Khan SMS. Impact of COVID-19 vaccination on menstrual cycle in women of reproductive age. Int J Gynaecol Obstet. 2023.

[66] Rastegar T, Feryduni L, Fakhraei M. COVID-19 vaccine side effects on menstrual disturbances among Iranian women. New Microbes New Infect. 2023;53:101114.

[67] Rodríguez Quejada L, Toro Wills MF, Martínez-Ávila MC, Patiño-Aldana AF. Menstrual cycle disturbances after COVID-19 vaccination. Womens Health (Lond). 2022;18:17455057221109375.

[68] Rogers A, Rooke E, Morant S, Guthrie G, Doney A, Duncan A, et al. Adverse events and overall health and well-being after COVID-19 vaccination: interim results from the VAC4COVID cohort safety study. BMJ Open. 2022;12(6):e060583.

[69] Saleh Alzahrani H, Ali Algashami S, Abdulaziz Alharkan A, Sultan Alotaibi N, Waseem Algahs N. The effect of COVID-19 vaccination on the menstrual cycle in female in Riyadh, Saudi Arabia. Saudi Pharm J. 2023;31(5):746-51.

[70] Sarfraz A, Sarfraz Z, Sarfraz M, Nadeem Z, Felix M, Cherrez-Ojeda I. Menstrual irregularities following COVID-19 vaccination: A global cross-sectional survey. Ann Med Surg (Lond). 2022;81:104220.

[71] Sualeh M, Uddin MR, Junaid N, Khan M, Pario A, Ain Q. Impact of COVID-19 Vaccination on Menstrual Cycle: A Cross-Sectional Study From Karachi, Pakistan. Cureus. 2022;14(8):e28630.

[72] Taşkaldıran I, Vuraloğlu E, Bozkuş Y, Turhan İyidir Ö, Nar A, Başçıl Tütüncü N. Menstrual Changes after COVID-19 Infection and COVID-19 Vaccination. Int J Clin Pract. 2022;2022:3199758.

[73] Velasco-Regulez B, Fernandez-Marquez JL, Luqui N, Cerquides J, Lluis Arcos J, Fukelman A, et al. Is the phase of the menstrual cycle relevant when getting the covid-19 vaccine? Am J Obstet Gynecol. 2022;227(6):913-5.

[74] Wali R, Alhindi H, Saber A, Algethami K, Alhumaidah R. The Effect of COVID-19 Vaccine on Women's Reproductive Health: A Cross-Sectional Study. Cureus. 2023;15(6):e40076.

[75] Wang S, Mortazavi J, Hart JE, Hankins JA, Katuska LM, Farland LV, et al. A prospective study of the association between SARS-CoV-2 infection and COVID-19 vaccination with changes in usual menstrual cycle characteristics. Am J Obstet Gynecol. 2022;227(5):739.e1-.e11.

[76] Wesselink AK, Lovett SM, Weinberg J, Geller RJ, Wang TR, Regan AK, et al. COVID-19 vaccination and menstrual cycle characteristics: A prospective cohort study. Vaccine. 2023;41(29):4327-34.

[77] Wong KK, Heilig CM, Hause A, Myers TR, Olson CK, Gee J, et al. Menstrual irregularities and vaginal bleeding after COVID-19 vaccination reported to v-safe active surveillance, USA in December, 2020-January, 2022: an observational cohort study. Lancet Digit Health. 2022;4(9):e667-e75.

[78] Woon EV, Male V. Effect of COVID-19 vaccination on menstrual periods in a prospectively recruited cohort. Front Reprod Health. 2022:1-5.

[79] Zhang B, Yu X, Liu J, Liu J, Liu P. COVID-19 vaccine and menstrual conditions in female: data analysis of the Vaccine Adverse Event Reporting System (VAERS). BMC Womens Health. 2022;22(1):403.

**
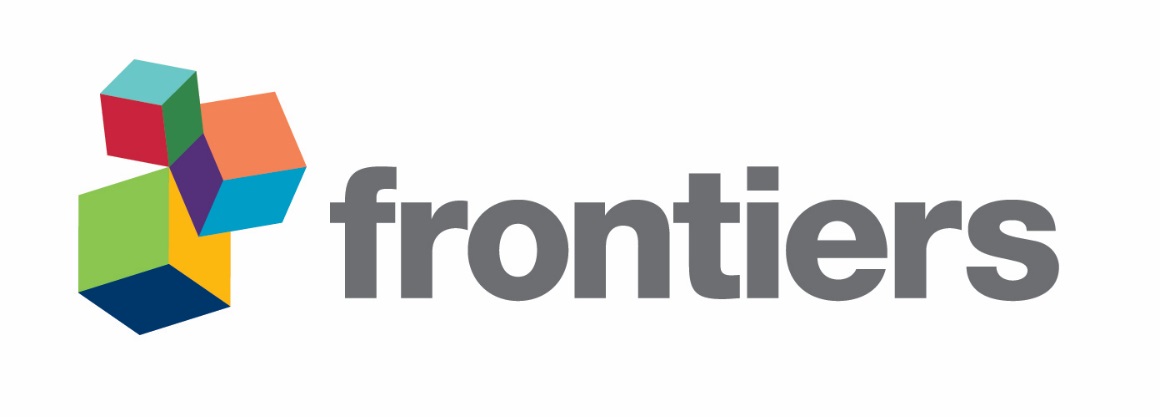
**
